# Beta Frequency Oscillations in the Subthalamic Nucleus Are Not Sufficient for the Development of Symptoms of Parkinsonian Bradykinesia/Akinesia in Rats

**DOI:** 10.1523/ENEURO.0089-19.2019

**Published:** 2019-10-21

**Authors:** Christina Behrend Swan, Derek J. Schulte, David T. Brocker, Warren M. Grill

**Affiliations:** 1 Department of Biomedical Engineering, Duke University, Durham, NC 27708; 2 Duke University School of Medicine, Durham, NC 27710; 3 Departments of Neurobiology and Neurosurgery, Duke University Medical Center, Durham, NC 27710

**Keywords:** β band oscillations, bradykinesia, Parkinson’s disease

## Abstract

Substantial correlative evidence links the synchronized, oscillatory patterns of neural activity that emerge in Parkinson’s disease (PD) in the beta (β) frequency range (13–30 Hz) with bradykinesia in PD. However, conflicting evidence exists, and whether these changes in neural activity are causal of motor symptoms in PD remains unclear. We tested the hypothesis that the synchronized β oscillations that emerge in PD are causal of symptoms of bradykinesia/akinesia. We designed patterns of stimulation that mimicked the temporal characteristics of single unit β bursting activity seen in PD animals and humans. We applied these β-patterned stimulation patterns along with continuous low-frequency and high-frequency controls to the subthalamic nucleus (STN) of intact and 6-OHDA-lesioned female Long–Evans and Sprague-Dawley rats. β-Patterned paradigms were superior to low-frequency controls at induction of β power in downstream substantia nigra reticulata (SNr) neurons and in ipsilateral motor cortex. However, we did not detect deleterious effects on motor performance across a wide battery of validated behavioral tasks. Our results suggest that β frequency oscillations (BFOs) may not be sufficient for the generation of bradykinesia/akinesia in PD.

## Significance Statement

We explored whether a causal link exists between the synchronized, 13–30 Hz (β band) oscillations that emerge in Parkinson’s disease (PD) and symptoms of bradykinesia/akinesia. The results provide not only for a better understanding of disease pathophysiology but also offer insights into the development of improved and novel treatments for PD. Our study suggests that β frequency oscillations (BFOs) are not causally related to bradykinesia/akinesia in PD.

## Introduction

The execution of voluntary movement relies on the coordinated generation, refinement, and relay of neural signals by a network of cortical and subcortical structures. In Parkinson’s disease (PD) deterioration of the dopaminergic nigrostriatal projection from death of substantia nigra compacta (SNc) neurons disrupts this network and engenders symptoms of bradykinesia/akinesia, rigidity, rest tremor, and postural instability (Dauer and Przedborski, 2003; Alves et al., 2008; Holtbernd and Eidelberg, 2012). Loss of nigral dopaminergic input to the striatum results in significant changes in neural firing rates and patterns including increases in burst and oscillatory firing, as well as excessive synchronization of firing within and across nuclei in the cortico-basal ganglia loop (Brown et al., 2001; Levy et al., 2002; Kühn et al., 2005). While the neural mechanisms underlying the motor symptoms of PD are unknown, particular attention has been given to synchronized, oscillatory neural activity occurring in a frequency band of 13–30 Hz, termed the β band, as these oscillations seem to be correlated with symptoms of bradykinesia in PD (Kühn et al., 2008). Indeed, treatments that improve bradykinesia, including levodopa administration or deep brain stimulation (DBS), also disrupt the β band oscillations that are seen throughout the cortico-basal ganglia loop, including in the subthalamic nucleus (STN; Levy et al., 2002; Kühn et al., 2006, 2008; Dorval et al., 2010; Delaville et al., 2015). While the origin of β oscillatory activity in PD is still unknown, the STN (Plenz and Kital, 1999; Gatev et al., 2006; Stein and Bar-Gad, 2013), the striatum (McCarthy et al., 2011), and the motor cortex (Yamawaki et al., 2008) have been postulated to be involved in the generation of β band oscillations in PD. Regarding the STN, STN DBS at 20 Hz in PD patients reduced finger tapping rates (i.e., increased bradykinesia) as compared to 0 Hz and 50 Hz DBS (Chen et al., 2007). Such observations suggest a causal link between STN β band oscillations and symptoms of bradykinesia and akinesia in PD. Conversely, animal models of PD demonstrated emergence of bradykinetic/akinetic symptoms before development of β band oscillations (Degos et al., 2009). Chronic administration of a dopamine receptor blocker to intact rats resulted in an immediate increase in akinetic symptoms, but an increase in β oscillatory power in primary sensorimotor cortex was not seen until the fourth day of treatment (Degos et al., 2009). If symptoms of bradykinesia/akinesia can be dissociated from β band oscillations, these oscillations may be an epiphenomenon.

We tested the hypothesis that a causal relationship exists between STN β band oscillations and symptoms of bradykinesia/akinesia in PD. We designed stimulation patterns to mimic the oscillatory burst firing seen in single STN neurons in human PD patients (Levy et al., 2002) and animal models of PD (Mallet et al., 2008). We applied these stimulation patterns to a biophysically-based computational model of the intact cortico-basal ganglia-thalamic loop (Kumaravelu et al., 2016) and assessed changes in β power in the activity of model globus pallidus internus (GPi) neurons. We then applied the stimulation patterns to the STN of healthy (intact) and parkinsonian (lesioned) rats and quantified entrainment of downstream neurons and effects on motor performance. The stimulation patterns increased β frequency power (BFP) in both the computational model and healthy rats. However, we did not detect deleterious effects of the patterns on rat motor performance. Our findings challenge the notion of a causal link between STN β band oscillations and symptoms of bradykinesia/akinesia in PD and suggest that STN β band oscillations are not sufficient for the development of these symptoms in PD.

## Materials and Methods

**Figure 1:**
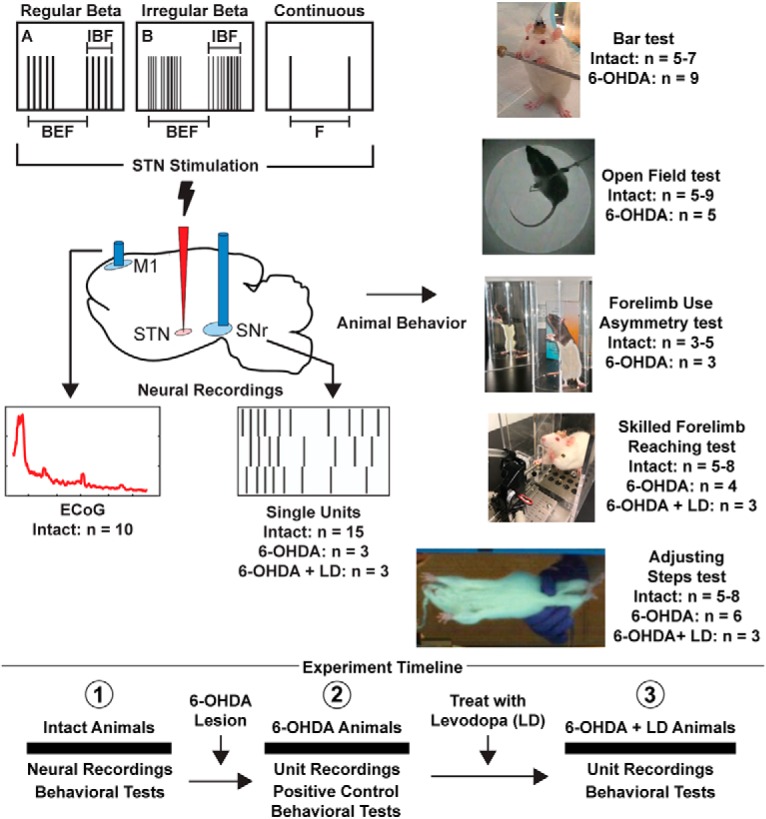
. Experimental design to assess the effects of betaergic DBS on neural activity and motor function. (1) Different temporal patterns of STN stimulation were applied in random order to intact rats. The effects on downstream SNr single units and M1 ECoG were recorded to quantify induced β. Rats performed a variety of behavioral tasks during betaergic stimulation to assess impact of stimulation on bradykinesia/akinesia. (2) Rats were then unilaterally lesioned via injection of 6-OHDA into the MFB. Betaergic stimulation patterns were again applied in random order, and the effect on SNr single units was recorded. The performance of 6-OHDA**-**lesioned rats was assessed in all behavioral tasks with and without 130-Hz stimulation. (3) Finally, 6-OHDA**-**lesioned rats were treated with levodopa, and the effect of betaergic stimulation patterns on SNr units was recorded. The performance of levodopa-treated 6-OHDA rats in the adjusting steps and skilled forelimb reaching task was quantified during different patterns of betaergic stimulation. LD = levodopa, IBF = Intra-Burst Frequency, BEF = Burst Envelope Frequency.

We first applied patterns of stimulation confirmed to induce β oscillatory power in a model cortico-basal ganglia circuit to the STN of intact rats. We used intact rats to apply our patterns to a “naive substrate,” as intact rats lack excessively synchronized endogenous β oscillations that would confound any BFP generated by our patterns (Fig. 1). In the second phase of our study we applied our patterns to 6-OHDA-lesioned animals treated with levodopa doses titrated to disrupt endogenous BFP. The goal of this approach was to exploit the changes in circuit dynamics that occur in PD while controlling the source of BFP. The Duke University Institutional Animal Care and Use Committee approved all animal care and experimental procedures. Animals were housed in Duke University Division of Laboratory Animal Resources managed housing with unrestricted access to food and water except as detailed below for food reward-motivated behavioral tasks.

### Chronic electrode implants

Anesthesia was induced in 10 female Long–Evans and 25 female Sprague Dawley rats (250–350 g) using a mixture of 7% sevoflurane and 2 l min^−1^ O_2_ and maintained during surgery using 2.5–3.5% sevoflurane in 1–1.5 l min^−1^ O_2_. Paw pinch withdrawal, heart rate, and peripheral capillary oxygen saturation were monitored to ensure appropriate anesthetic depth. All cranial implants were performed using a Kopf stereotaxic frame with stereotaxic coordinates determined from a rat brain atlas (Paxinos and Watson, 2005). Rats were implanted unilaterally with a 23-gauge cannula in the medial forebrain bundle [MFB; from bregma: anterior-posterior (AP) –2.0 mm, medial-lateral (ML) ±2.0 mm; from brain surface: dorsal-ventral (DV) –7.0 mm]; a four-channel stimulating microelectrode array (MEA) in the STN (AP –3.6 mm, ML ±2.6 mm; from brain surface: DV: –6.8 to –7.2 mm); and a 16-channel recording MEA in the substantia nigra reticulata (SNr; AP –5.8 mm, ML ±2.3 mm; from brain surface: DV –6.8 to –7.2 mm). The STN stimulating MEA was comprised of 2 × 2 75 μm 10-kΩ platinum-iridium (Pt-Ir) electrodes with an interelectrode spacing of 300 μm (Microprobes for Life Science). Intraoperative recordings using a single 0.5-MΩ tungsten microelectrode were conducted to confirm the stereotaxic location of the STN before placement of the 2 × 2 MEA in the STN. The SNr recording MEA was comprised of 4 × 4 125-μm 0.5-MΩ Pt-Ir electrodes with interelectrode spacing of 250 μm (Microprobes). Intraoperative recordings were performed as the 4 × 4 array was advanced to confirm the depth of SNr. All unilaterally placed implants were localized to the same hemisphere. Nine stainless steel bone screws were affixed to the skull. Three of these bone screws were placed over bilateral primary motor cortex (M1; AP +2.5 mm, ML ±2.5 mm) and unilateral primary somatosensory cortex (S1; AP –1.0 mm, ML 3.0 mm). Electrocorticogram (ECoG) signals were measured by wiring these bone screws to an additional connector in the headcap. A bone screw over the cerebellar cortex served as a reference for neural recordings. Dental acrylic was used to secure all implants to the bone screws. Animals recovered for one week before any additional procedures were performed.

### Stimulation parameters

We designed different temporal patterns of electrical stimulation to mimic the oscillatory burst firing seen in PD patients and parkinsonian animal models, including both regular and irregular “β” bursting paradigms. We applied three regular and one irregular β bursting paradigms, low (15, 20, 25 Hz) and high (225 Hz) frequency continuous stimulation patterns, which served as “control” patterns to the bursting paradigms, during behavioral tasks and neural recordings, yielding a total of eight experimental stimulation patterns. The regular β bursting paradigms were non-varying patterns with fixed burst envelope frequency (BEF), intraburst frequency (IBF), and number of pulses per burst (Fig. 1). We selected BEFs of 15, 20, and 25 Hz, which corresponded approximately to the peak frequencies of β oscillatory activity of STN neurons seen in parkinsonian non-human primates (Moran et al., 2012), human PD patients (Kühn et al., 2008; Bronte-Stewart et al., 2009; Eusebio and Brown, 2009), and 6-OHDA-lesioned rats (Sharott et al., 2005), respectively. For each BEF we used an IBF of 225 Hz and five pulses per burst based on analysis of published recordings of STN single unit burst firing in PD patients (Levy et al., 2000, 2002). An IBF of 225 Hz is consistent with IBFs in STN neurons of PD patients, which have been seen to range from 75 to over 200 Hz (Birdno and Grill, 2008; Gale et al., 2009; Welter et al., 2011).

We designed the irregular β bursting paradigm through computational optimization using a genetic algorithm (Brocker et al., 2017). The irregular β bursting paradigm was parameterized as a binary string. Each element represented a time bin of 1.5 ms. A “0” represented no stimulation and a “1” represented stimulation. The optimization objective was to determine the appropriate arrangement of 1s and 0s that maximized β band oscillatory power in the firing of model GPi neurons in the computational model of the intact basal ganglia (See section Computational model). The fitness function was equal to the maximum power in the β band of GPi neuron firings averaged over time and across GPi cells. The most “fit” patterns were mated using fitness proportionate selection. The resulting stimulation train had a BEF of ∼15 Hz, an IBF of ∼700 Hz, a varying number of pulses per burst, and an average number of total pulses per second of 225 (Fig. 1).

All stimulation patterns were comprised of charged-balanced, symmetric biphasic pulses of 90 μs/phase. MATLAB (R2009a, The MathWorks) was used to digitize stimulation pattern templates and drive a voltage-to-current stimulus isolator (A-M Systems). Bipolar stimulation was delivered through the stimulating MEA to the STN. Amplitudes for each animal ranged from 20 to 150 μA. The maximum stimulation amplitude chosen for each animal was the amplitude at a continuous stimulation frequency of 130 Hz that induced circling contralateral to the hemisphere to which stimulation was applied but minimized additional motor effects such as paw tremor or rearing. For behavioral studies, a single amplitude, the maximum determined for each animal, was applied for each stimulation pattern. For neural recording studies with stimulation, the applied amplitudes ranged from a submotor threshold amplitude, usually 20–25 μA, to the maximum amplitude determined for that animal. In summary, we applied eight stimulation patterns across a range of amplitudes during neural recordings and a variety of behavioral tasks. Three regular bursting and one irregular bursting pattern were designed to mimic STN unit bursting activity seen in PD humans and rats. Three low-frequency patterns matching the BEFs of the regular bursting patterns (15, 20, 25 Hz) were applied to serve as controls that had β spectral frequency but no bursts. A high-frequency pattern matching the IBF of the regular bursting patterns (225 Hz) was applied as an additional control.

### Computational model

The regular bursting, irregular bursting, and continuous frequency stimulation patterns were applied to the STN model neurons in a biophysically-based model of the cortico-basal ganglia-thalamic network (Kumaravelu et al., 2016). The model was comprised of a network of single compartment Hodgkin-Huxley type neurons representing regular spiking excitatory neurons and fast-spiking interneurons in cortex; direct (dopamine-type 1 receptor dominant) and indirect (dopamine-type 2 receptor dominant) medium spiny neurons of the striatum; STN; globus pallidus externus (GPe); GPi; and thalamus, and 59 cells of each cell type were included in the model. Multiple amplitudes were tested to span the range from no STN cell activation to activation of all model STN neurons. The pulse width of each monophasic pulse was fixed at 180 μs. Simulations were implemented in MATLAB R2014a with equations solved using the forward Euler method with a time step of 0.025 ms and a simulation length of 10 s (Kumaravelu et al., 2016).

The output measure was the peak power of β band oscillatory activity averaged across all model GPi neurons. The averaged peak β band power was calculated by first generating the average multi-taper spectrogram of the spike times of all GPi neurons using Chronux (chronux.org) with MATLAB R2014a. A sliding window of 1 s and a step size of 0.1 s was used. Second, at each time point of the averaged spectrogram, the peak power between 13 and 30 Hz was extracted. Finally, all maximum power values across time were averaged to generate the average maximum β band power for a given stimulation train pattern and amplitude.

### Code accessibility

Our model of the cortico-basal ganglia-thalamic network is available for download on Model DB, accession number 206232.

### Behavioral tests before unilateral 6-OHDA lesion

Healthy (dopamine-intact) rats performed a variety of behavioral tasks after recovery from chronic electrode implantation to assess for induction of bradykinetic/akinetic symptoms by stimulation of the STN with any of the patterns. To mitigate that a single metric from one behavioral task may alone not be sensitive enough to detect stimulation induced symptoms, we used a constellation of previously validated quantitative behavioral outcomes. These tasks are widely used to assess the degree of bradykinetic/akinetic impairment in rodent models of PD, are sensitive to varying degrees of SNc dopaminergic cell loss, and should detect changes in motor function induced by applied β-patterned stimulation paradigms. The stimulation patterns tested and the number of animals that performed each behavioral task are detailed in Table 1.

**Table 1.** Stimulation patterns tested in model simulations, healthy (intact) animals, and 6-OHDA-treated animals

Pattern	Model	Bar test	Open field test	Adjusting steps test	Forelimb use asymmetry test	Skilled forelimb reaching test
Intact animals						
No stimulation	X	7	9	8	5	8
Irregular β	X	5	5	7	5	5
B15 IBF225	X	5	6	7	3	6
B20 IBF225	X	5	5	5		5
B25 IBF225	X	5	6	7	3	6
Regular 15 Hz	X	7	8	6	3	5
Regular 20 Hz	X	5	5	5		5
Regular 25 Hz	X	5	6	7		6
Regular 225 Hz	X	7	9	7	3	5
6-OHDA-treated animals						
No stimulation	X	9	5	6	3	4
Regular 130 Hz		9	5	3	3	3
6-OHDA levodopa + stimulation experiments						
Levodopa (LD)				3		3
LD + irregular β				3		3
LD + B15 IBF225				3		3
LD + B20 IBF225				3		3
LD + B25 IBF225				3		3
LD + regular 15 Hz				3		3
LD + regular 20 Hz				3		3
LD + regular 25 Hz				3		3
LD + regular 225 Hz				3		3

### Bar test

The bar test detects forelimb akinesia, which develops prominently in the 6-OHDA rat model of PD with >90% dopaminergic SNc cell loss (Duty and Jenner, 2011). When placed in an abnormal upright posture with forelimbs gripping a bar typically 5–10 cm above the ground, 6-OHDA-lesioned rats will take longer to release the bar than unlesioned rats (Duty and Jenner, 2011). The goal in using this behavioral task was to determine whether the unilaterally applied β-patterned stimulation paradigms preferentially caused generation of contralateral forelimb bradykinetic or akinetic symptoms as compared to no stimulation and continuous stimulation controls. For this test, dopamine-intact animals were placed in a 36 × 24 cm clear plastic chamber with a 0.5-cm diameter bar 10 cm above the ground in a dim room. Animals received unilateral STN stimulation at the maximum amplitude determined for that animal. An experimental session alternated between blocks of no stimulation and blocks of stimulation such that each stimulation block was bracketed by a no stimulation block. Each block was comprised of “pre-trial” and “trial” periods. For a given block, the animal experienced a pre-trial period of 3 min of either no stimulation or a specific pattern of stimulation. After 3 min elapsed, the trial period began. If the block was a stimulation block, stimulation continued uninterrupted from the pre-trial period through the trial period. The animal was placed such that it was standing upright on its hind paws while its forepaws gripped the bar (see Fig. 6*A*). The total time each paw spent on the bar was measured as a function of stimulation paradigm. A trial ended after either three placements on the bar were quantified or 300 s elapsed. The order in which stimulation patterns were presented was randomized between experimental sessions. An experimental session lasted 1–2 h depending on an animal’s performance. For each animal, the total time each paw spent on the bar was averaged for each stimulation pattern. An increase in length of time on bar for the paw contralateral to the stimulated hemisphere as compared to the ipsilateral paw and the no stimulation result was interpreted as generation of bradykinetic/akinetic symptoms.

### Open field test

The open field test assesses spontaneous locomotor activity and is sensitive enough to discriminate between motor deficits caused by complete (>90% SNc dopaminergic cell loss) and incomplete (∼70% SNc dopaminergic cell loss) lesions in the 6-OHDA rat model of PD (Carvalho et al., 2013). 6-OHDA-lesioned animals exhibit less motor activity than intact animals in an open field (Carvalho et al., 2013). The goal was to determine whether unilaterally applied β-patterned stimulation paradigms preferentially caused a decrease in movement speed or a decrease in the number of movement initiation episodes as compared to no stimulation and continuous stimulation controls. Dopamine-intact rats were placed in a dark 20 cm in diameter cylinder, and an infrared camera captured all movements (see Fig. 7*A*). Animals received unilateral STN stimulation at the maximum amplitude determined for that animal. An experimental session alternated between blocks of no stimulation and blocks of stimulation such that each stimulation block was bracketed by a no stimulation block. Each block was 3 min long. An experimental session was limited to 60 min to minimize overall experiment duration. As such, during each experimental session, one to two β bursting paradigms in addition to the corresponding low and high-frequency continuous control patterns were applied. Additional experimental sessions were performed after a rest period of ∼5–7 d to avoid habituation of the animal to the chamber. The order in which stimulation patterns were presented was re-randomized between experimental sessions. Videos were analyzed using TopScan version 2.0 (CleverSystems, Inc.) and MATLAB. Average linear speed, average number of pauses per second, and average pause length were measured, and results were normalized for each animal to the no stimulation results to facilitate comparison across animals. A significant decrease in average linear speed and a significant increase in average pause length and average number of pauses per second as compared to the no stimulation results was interpreted as generation of bradykinesia/akinesia.

### Adjusting steps test

The adjusting steps test assesses forelimb akinesia in dopamine-depleted rats and can detect a forelimb motor deficit at a striatal dopamine depletion levels of 80% or greater (Chang et al., 1999). When suspended vertically such that the body weight is supported by the forelimbs, a 6-OHDA-lesioned rat will drag its affected forelimb rather than making adjusting steps to support its weight when dragged backwards or laterally (Chang et al., 1999; Fleming, 2009; Glajch et al., 2012). The goal was to determine whether unilaterally applied β-patterned stimulation paradigms preferentially caused an increase in akinesia in the contralateral forelimb as compared to no stimulation and continuous stimulation controls. Dopamine-intact rats were gripped around the hips to immobilize the hindlimbs and suspended vertically on a 77 × 16 cm glass plank such that the animal supported its weight through its forepaws (see Fig. 8*A*). A video camera positioned below the glass plank recorded all forelimb movements. Animals were dragged backwards, and the number of backward steps the animal made with each forepaw along the length of the glass plank was recorded. The animal performed three to five trials of this task. The animal then was returned to its home cage and received unilateral STN stimulation at the maximum amplitude determined for that animal for 5 min. While STN stimulation continued, each animal performed three to five additional trials of this task. A single pattern of stimulation was applied during an experimental session, which typically lasted 30 min, and animals were given at least 3 h of rest in between experimental sessions. A decrease in forelimb adjusting steps for the forelimb contralateral to the stimulated hemisphere as compared to the ipsilateral forelimb and the no stimulation result was interpreted as generation of bradykinesia/akinesia.

### Forelimb use asymmetry test

The forelimb use asymmetry test assesses forelimb akinesia in dopamine-depleted rats, and performance in this task correlates with the amount of striatal dopamine depletion (Connor et al., 1999; Fleming, 2009). When placed in a narrow cylinder, 6-OHDA-lesioned rats will avoid using the affected forelimb during vertical exploration of the cylinder (Schallert et al., 2000; Fleming, 2009). The goal was to determine whether unilaterally applied β-patterned stimulation paradigms preferentially caused a decrease in limb use preference in the contralateral forelimb during vertical exploration as compared to no stimulation and continuous stimulation controls. Dopamine-intact rats were placed in their home cage for 1 h in a dark room to acclimate to the environment. After 1 h, rats received either no stimulation or unilateral STN stimulation with a single pattern for 5 min. Rats were then placed in a 20 cm in diameter × 30 cm high clear cylinder for 5–10 min (see Fig. 9*A*). If stimulation was applied in a given experimental session, it continued for the full duration of the task. Animals were videotaped during the task, and mirrors were used to capture a 360° view of animal exploration of the cylinder. During rears the numbers of single and bilateral forepaw touches to the cylinder wall were quantified. Additionally, on descending from a rear, the rat’s preference for using one or both forepaws to support its weight was quantified. Animals were given 7 d of rest between experimental sessions to diminish habituation to the cylinder. A decrease in use of the forelimb contralateral to the stimulated hemisphere on vertical exploration and landings as compared to the ipsilateral forelimb and the no stimulation result was interpreted as generation of bradykinesia/akinesia.

### Skilled forelimb reaching test

A skilled forelimb reaching task was conducted using the Vulintus MotoTrak System (Hays et al., 2013). Animals were placed in a 36 × 24 × 16 cm acrylic chamber with a narrow slot at one end positioned so as to restrict performance to a specific forelimb. A lever on a motorized track was positioned at or just outside of the slot, and animals were trained to depress the lever twice within a certain time window to receive a food reward (see Fig. 10*A*). Impaired animals demonstrate longer interpress intervals, fewer presses per trials, and fewer successes (Hays et al., 2013).

The goal was to determine whether unilaterally applied β-patterned stimulation preferentially caused impairment in the forelimb contralateral to the stimulated hemisphere during lever presses as compared to no stimulation and continuous stimulation controls. Dopamine-intact rats were restricted to 12 g of food per day until their body weight was between 85% and 90% of their free feeding weight. During an experimental session, a rat was placed in its home cage in a dark room and received unilateral STN stimulation at the maximum amplitude determined for that animal for 5 min. The rat then was placed in the experimental chamber while stimulation continued. The animal was allowed 30 min in the experimental chamber to perform the task. A successful trial was defined as two lever presses within a 0.5-s window with the lever positioned 1.0 cm outside of the chamber. Interpress interval, mean press duration, initiation of press to hit peak latency, and success rate to a successful trial were calculated. A maximum of two experimental sessions were performed per day, and animals rested for at least 3 h between sessions. Sessions without stimulation were conducted on an identical timeline to those with stimulation. An increase in interpress interval, mean press duration, and initiation of press to hit peak latency and a decrease in success rate were interpreted as generation of bradykinesia/akinesia.

### 6-OHDA lesioning

Eleven animals were rendered hemi-parkinsonian through unilateral administration of the neurotoxin 6-hydroxydopamine hydrobromide (6-OHDA, Sigma-Aldrich) via the MFB cannula to evoke unilateral degeneration of dopaminergic neurons in the nigrostriatal pathway (Tieu, 2011). As 6-OHDA also will selectively destroy noradrenergic neurons, 30 min before 6-OHDA administration animals were pre-treated with intraperitoneal injections of 5-mg/kg desipramine (Sigma-Aldrich) to protect nonadrenergic neurons and 50-mg/kg pargyline (Sigma-Aldrich) to inhibit monoamine oxidase activity (McConnell et al., 2012; So et al., 2012). Anesthesia was induced using a mixture of 7% sevoflurane in 2 l min^−1^ O_2_ and maintained using 2.5–3.5% sevoflurane in 1–1.5 l min^−1^ O_2_. Animals were positioned in a Kopf stereotactic frame for intracerebral injection. Immediately before infusion, 5-mg 6-OHDA was dissolved into 2 mL 0.02% ascorbic saline (Sigma-Aldrich) stored at 4°C to produce a final concentration of 10 mM. Ten microliters of 6-OHDA solution were administered through the MFB cannula using a Hamilton syringe at a rate of 2 μl/min. Animals were given one week to recover. Animals that did not exhibit unilateral motor symptoms after the recovery period were re-infused with 6-OHDA a maximum of two additional times.

### Methamphetamine-induced circling test

One week after injection of 6-OHDA, each rat was injected with a methamphetamine solution (Sigma-Aldrich) at a concentration of 1.5–2.5 mg/kg intraperitoneally and placed in a 20 cm in diameter × 30 cm high cylinder within a dark chamber. Animals with severe unilateral lesions of dopaminergic pathways will circle ipsilateral to the lesion after administration of methamphetamine (Ungerstedt and Arbuthnott, 1970). The activity of the animal was monitored using an infrared lamp and camera for 2 h, and during this time blocks of high-frequency stimulation were applied for 60 s with 120 s of rest between each stimulation pattern (McConnell et al., 2012). TopScan version 2.0. was used to determine the position of each animal within the cylinder for each video frame. From this information, the angular velocity of each animal with and without stimulation was calculated using MATLAB. Based on previous histologic analysis, animals that circled at a rate of at least 3 turns/min with no stimulation were deemed to have had >90% loss of dopaminergic neurons in the SNc (Ungerstedt and Arbuthnott, 1970; Fang et al., 2006; So et al., 2012), which was defined as a successful lesioning procedure. Animals that did not meet this criterion were re-lesioned up to two additional times.

### Post-lesion behavioral positive controls

After confirmation of successful lesion via the methamphetamine-induced circling test, baseline performance and performance with continuous 130-Hz stimulation were assessed in the bar test, the open field test, the adjusting steps test, the forelimb use asymmetry test, and the skilled forelimb reaching test. The amplitude for 130-Hz stimulation was consistent with that used for each animal before the lesioning procedure. Assessing motor performance under these conditions provided a threshold for generation of parkinsonian symptoms through use of β-patterned stimulation for each behavioral task. The number of animals that performed each positive control task can be found in Table 1.

### Post-lesion β-patterned stimulation behavioral assessments

Rats were pre-treated with 15 mg/kg of benserazide hydrochloride (intraperitoneal; Sigma-Aldrich), a peripheral DOPA decarboxylase inhibitor, and L-3,4-dihydroxyphenylalanine methyl ester hydrochloride (levodopa; 15–19 mg/kg, i.p., Sigma-Aldrich). Doses of levodopa were selected for each animal that disrupted cortical M1 power ipsilateral to the lesion (See section Neuronal recordings after unilateral 6-OHDA lesion) but did not make the animal dyskinetic. The adjusting steps test and the skilled forelimb reaching task were administered to assess performance for a drug only baseline and during β-patterned and control stimulation. The patterns assessed are detailed in Table 1.

### Neuronal recordings

Sixteen single unit channels were recorded from the SNr, and three ECoG channels, M1 bilaterally and S1 from the hemisphere contralateral to STN implant, were recorded simultaneously using a multichannel acquisition processor system (Plexon, Inc.). The rat was placed in an open top chamber within a Faraday cage, and recordings were performed while the rat was awake and freely roaming the chamber. The three ECoG channels were also recorded after 6-OHDA lesion and used to titrate doses of levodopa as described below. The voltage signal used to drive the stimulus isolator was recorded simultaneously with the neural signals on an analog input channel to enable precise time-locking of the stimulation input to the neural recordings in subsequent analyses. For single unit recordings gain and filter settings were: gain = 20,000, filter = 150 Hz to 8.8 kHz, sampling rate = 40 kHz, and for LFP recordings, gain and filter settings were: gain = 2500–5000, filter = 150 Hz to 8.8 kHz, sampling rate = 20 kHz.

### Neuronal recordings before unilateral 6-OHDA lesion

Recording sessions were 3–4 h in length, and all patterns listed in Table 1 were applied during an experimental session. A single pattern block consisted of 90–120 s of no stimulation, 90–120 s of applied stimulation, and 90–120 s of no stimulation while single units were recorded from SNr. ECoG also was recorded simultaneously from bilateral M1 and contralateral S1. Each pattern was applied at three to four amplitudes, ranging from 25 μA to the highest tolerated amplitude for each animal. The order in which patterns and amplitudes were presented was randomized. Each animal participated in up to four recording sessions. Pattern and amplitude presentation were re-randomized between sessions.

### Neuronal recordings after unilateral 6-OHDA lesion

**Figure 2.**
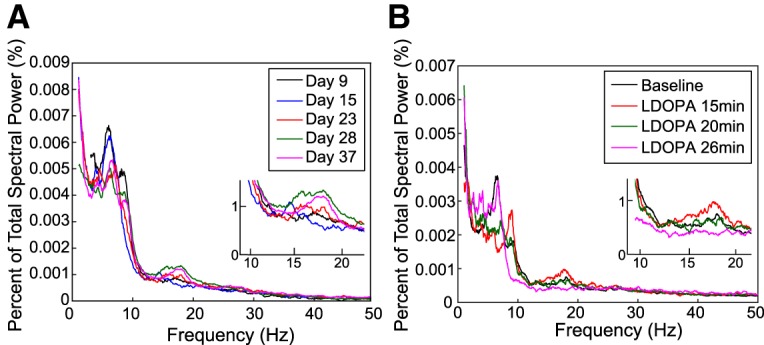
Sample post-6-OHDA lesion ipsilateral M1 ECoG recordings. Insets show a magnified view of β frequency range results. ***A***, Progressive increase in ipsilateral M1 β frequency activity as a function of days post-6-OHDA lesion. ***B***, Disruption of ipsilateral M1 β frequency activity as a function of time from injection of levodopa (LDOPA).

Two types of recording sessions were conducted. Although unilateral motor impairment can be detected shortly after 6-OHDA lesion increases in BFP appear to emerge after presentation of motor symptoms (Degos et al., 2009). We were able to detect β frequency peaks in ipsilateral M1 ECoG approximately three weeks post-lesion (Fig. 2*A*). Before emergence of this peak, stimulation and SNr unit recording sessions were performed in a manner identical to the recordings done in intact rats. Each animal participated in up to two experimental sessions. Pattern and amplitude were re-randomized between experimental sessions.

After emergence of a β peak in ipsilateral M1 ECoG, different doses of levodopa were administered during different recording sessions until sustained depression of ipsilateral M1 BFP was observed (Fig. 2*B*). As peak drug effect in the rat lasts approximately 2 h (Putterman et al., 2007), stimulation and SNr unit recording sessions then proceeded as without drug pre-treatment but were executed only at the maximum amplitude to allow for all patterns to be applied within the same recording session.

### Single unit recording analysis

Single units were sorted online using SortClient and were classified further using Offline Sorter (Plexon, Inc.). Timestamps of unit activity were imported into MATLAB for analysis. Interspike interval (ISI) histograms of unit activity were calculated for the baseline and stimulation periods. Artifact timestamps were extracted from the recorded voltage input signal and used to calculate interpulse interval (IPI) histograms and peri-stimulus time histograms (PSTHs). A virtual PSTH was calculated for the pre-stimulation time period by shifting the artifact timestamps to align with the beginning of the pre-stimulation recording period. Blanking periods of 0.7 ms before through 2 ms after the artifact timestamp were applied to eliminate the possibility of a portion of the artifact wave form being counted as a unit timestamp. These blanking periods were applied to the virtual PSTHs, as well. For burst stimulation patterns, PSTHs were aligned to the last pulse in each burst to assess activity in the interburst interval. A bin width of 0.2 s was used to generate the bin axes for both virtual and stimulation PSTHs. The change in unit activity from pre-stimulation baseline was determined by transforming the stimulation PSTH bin counts to z-scores relative to the virtual PSTH bin counts according to the following formula, where *i* represents a single stimulation PSTH bin value:

$$Z_{stim}(i)=\frac{\left(PSTH_{stim}\left(i\right)-\bar{PSTH_{virtual}}\right)}{\sigma_{virtual}}.$$

**Figure 3.**
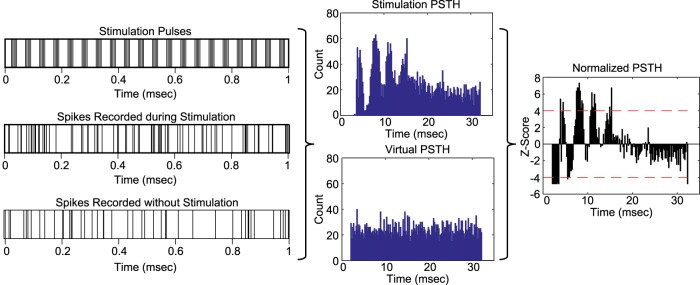
Calculation of normalized PSTH. Artifact timestamps were used to create a PSTH for the stimulation period and a virtual PSTH for the pre-stimulation period. Bin counts for the stimulation PSTH were converted to z-scores using the virtual PSTH bin counts to determine statistically significant changes in activity due to applied STN stimulation.

A threshold of four standard deviations from the mean (*Z_stim_(i)* ≥ 4, *p* < 0.001) was chosen to distinguish strong, statistically significant changes in unit activity from the pre-stimulation baseline in the normalized stimulation PSTH (Fig. 3).

To determine whether single unit activity in the SNr was entrained to stimulation in the STN, the excitatory effective pulse fraction (eEPF) was calculated using the normalized PSTH (Agnesi et al., 2015). The EPF is a ratio (range: 0–1) that relates the number of single unit firings evoked by a stimulus pulse at a consistent latency within the IPI to the number of unit firings evoked by a “virtual” stimulus pulse at the same latency during a baseline period. This latency within the IPI is referred to as a phase and represents at least two consecutive time bins with a statistically significant increase in single unit activity from the baseline period. The eEPF was designed for a stimulation site and recording site linked by a glutamatergic monosynaptic projection, as are the STN and SNr, and is calculated as follows for each identified IPI phase (Agnesi et al., 2015):

$$eEPF=\frac{pfs-pfsb}{number\;of\;stimulus\;pulses-pfsb},$$

where pfs represents the number of stimuli during the stimulation period followed by a single unit spike and pfsb represents the number of shifted stimulus pulses followed by a single unit spike during the pre-stimulation period. An eEPF was calculated for all distinct IPI phases of the normalized stimulation PSTH with statistically significant increases in unit activity from the pre-stimulation period (Fig. 3). If multiple distinct IPI phases were present in a single normalized stimulation PSTH, then the eEPFs for these phases were averaged to assess the overall effect of stimulation pattern and amplitude.

The change in BFP in SNr unit activity as a result of STN stimulation was quantified through calculation of multi-taper spectra using the Chronux data package for MATLAB. Spectra were calculated for both the pre-stimulation and stimulation time periods using averaging over 15- to 20-s windows (six time segments per recording period), five Slepian data tapers, a bandpass range of 1–58 Hz, and 0.04-Hz frequency resolution. To account for the effect of amplifier blanking, an individualized blanking period was determined for each unit. Using the lowest amplitude stimulation recording for each unit, a PSTH was created without pre-determined blanking and the length of time between a stimulation pulse and the first unit spike was found. This individualized blanking period was then imposed on the pre-stimulation recording for each unit; any spikes occurring within this time period following a virtual pulse were deleted. These blanking periods ranged from 0.6 to 2 ms and avoided introduction of artificial BFP into the spectra. After calculation of pre-stimulation and during stimulation spectra, the percentage of total power in the β band was quantified. Each power value was divided by the sum of all power values to convert it to a fraction of total power. The percentage of total power in the β band was then equal to the sum of each scaled power value in the range of 13–30 Hz. The difference between the percentage of total power in the β band with and without stimulation for each amplitude, frequency pattern, and single unit was determined.

### ECoG recording analysis

Three ECoG channels–bilateral M1 and S1 contralateral to the implanted STN–were recorded during stimulation in ten intact animals. Our goal was to quantify the amount of BFP induced by stimulation in ipsilateral M1 ECoG given the connections between STN and motor cortex (Gatev et al., 2006; Delaville et al., 2015). While projections between ipsilateral primary somatosensory cortex and STN have been identified in the rat, contralateral projections have not been identified (Canteras et al., 1988). To distinguish inducement of physiologic β power from the effect of stimulation artifact, the effects seen in ipsilateral M1 ECoG were referenced to contralateral S1 ECoG data as described below.

Ipsilateral M1 and contralateral S1 continuous data were imported into MATLAB and divided into pre-stimulation and with stimulation segments. ECoG segments recorded during stimulation were high-pass filtered using a three-pole Butterworth filter with a 2-Hz cutoff frequency. Stimulus artifacts were digitally blanked via linear interpolation from 0.1 to 1.5 ms after the start of a stimulus pulse. The data were then divided into segments of repeating IPIs and averaged to determine an average evoked response to stimulation. This average evoked response was subtracted from the overall dataset to reduce spectral power at the stimulation frequencies (Brocker et al., 2017). Finally, the data again were bandpass filtered using a three-pole Butterworth filter between 2 and 100 Hz. ECoG segments recorded before stimulation underwent only both rounds of filtering.

Multi-taper spectra were calculated for ipsilateral M1 and contralateral S1 ECoG channels for both pre-stimulation and during stimulation time segments. Spectra were calculated using averaging over 5-s windows, three Slepian data tapers, and a bandpass range of 3–55 Hz. As with the single unit spectra, the percentage of total power in the β band was quantified for both pre-stimulation and during stimulation spectra. The difference between the percentage of total power in the β band with and without stimulation for each amplitude and stimulation pattern was calculated for both ipsilateral M1 and contralateral S1 spectra. Finally, the percentage of total power in the β band during stimulation for contralateral S1 ECoG was subtracted from the percentage of total power in the β band during stimulation for ipsilateral M1 ECoG (See section Pre-6-OHDA ECoG Recordings).

### Histology

Histologic analysis was conducted to determine the location of all implanted electrode arrays as well as the extent of the 6-OHDA lesion. Each animal was deeply anesthetized with urethane (1.8 g/kg, i.p.) and transcardially perfused with cold PBS followed by 10% formalin. The head was removed and post-fixed in 10% formalin overnight at 4°C. The following day, the brain was removed and placed in a 30% sucrose solution and stored at 4°C until it sank, usually ∼48 h. The left hemisphere was dyed to assist in hemispherical identification of brain slices. Brains were then placed in optimal cutting temperature compound (OCT, Tissue Tek) and frozen to –80°C. A cryostat at approximately –20°C was used to cut 50-μm serial coronal slices.

**Figure 4.**
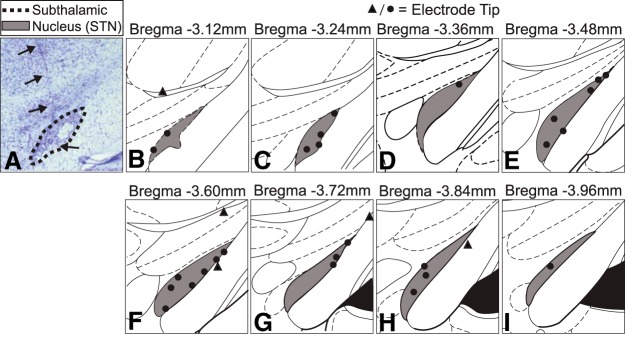
Histologic analysis of STN electrode positions. ***A***, Light microscopy image of cresyl violet stained brain slice. Dashed line denotes outline of STN. Arrows indicate electrode track. ***B****–****I***, Locations of STN electrode tips. Each circle (●) or triangle (▲) indicates the location of the deepest electrode tip for one animal. Only animals with electrode tips in the STN (●) were included in analysis. Animals with electrode tips outside of the STN (▲) were excluded from analysis.

To identify implanted electrode tip locations, a cresyl violet stain was used. Brain slices were rinsed in PBS, mounted onto microscope slides, and left to dry overnight. Mounted slices were de-fatted by placing slides in forward and then backward through a series of solutions for 3 min each: distilled water, 70% ethanol, 95% ethanol × 2, 100% ethanol × 2, Histoclear × 2. Slides were then stained in a 0.1% cresyl violet solution (Sigma-Aldrich) for ∼30 min, dried, and coverslipped. Slides were visually inspected using a light microscope to locate electrode tips. Rats were excluded from analysis if stimulating electrode tips were not located within the STN (Fig. 4).

To visualize the extent of the 6-OHDA lesion, a fluorescent immunostaining protocol was used. Slices were rinsed three times in a 1× PBS solution and incubated for 1 h at 4°C in a 1× PBS-based solution containing 8% normal goat serum (Jackson ImmunoResearch) and 1% Triton X-100 (VWR). Slices were rinsed three times in 1× PBS for 5 min each and then incubated overnight at 4°C in a primary 1× PBS-based solution containing 2% normal goat serum and 0.2% anti-tyrosine hydroxylase (anti-TH; monoclonal mouse IgG1; 1:500; Sigma-Aldrich). Slices were again rinsed three times in 1× PBS for 5 min each and then incubated for 1 h in a secondary 1× PBS-based solution containing 2% normal goat serum, 0.5% Triton X-100, and 0.2% Alexa Fluor 594 goat anti-mouse IgG1 (Life Technologies). Slices were then rinsed three times in 1× PBS for 5 min each and coverslipped with SouthernBiotech DAPI-Fluoromount-G (Fisher Scientific).

### Experimental design and statistical analyses

All animal data are expressed as mean ± SE with *n* = the number of animals and reported for each behavioral test in Table 1. To determine significance in behavioral performance metrics, animal data were analyzed using repeated measures ANOVA (RMANOVA), unpaired *t* tests, and paired *t* tests as appropriate with fixed factors including paw, pattern, amplitude, lesion state, and/or drug state depending on the particular behavior test and animal ID as the random variable. The Shapiro-Wilk test was used to test normality of the data, which was found to be normal or near normal. Given the robustness of ANOVA both to distributions with significant departures from normality and to small sample sizes (Pearson, 1931; Normal, 2010; Blanca et al., 2017), we used parametric statistics for the majority of our data. Wilcoxon rank sum tests were used to analyze censored data (bar test). Interpretation of the effects of stimulation on neural data were made using ANOVA with fixed factors including pattern, amplitude, lesion state, and/or drug state as appropriate. Where ANOVAs and RMANOVAs were found to be significant, *post hoc* tests to distinguish differences among factor levels were the Student’s *t* test if a factor had only two levels or the Tukey’s honest significant difference (HSD) if a factor had greater than two levels. The alpha level chosen for statistical significance was 0.05.

## Results

### Histologic analysis of electrode locations and 6-OHDA lesions

Verification of stimulation electrode tip locations in STN is shown in Figure 4. Only animals with electrode tips located in the STN were included in behavioral and neural analyses. For 3/35 animals, brains were not available for histologic processing. Previously recorded videos of behavioral response to 130-Hz stimulation were used to determine whether to include these animals in subsequent analyses, as a contralateral turning response to high-frequency stimulation correlates very strongly with successful targeting of the STN (So et al., 2012).

### Effects of β-patterned stimulation: model cortico-basal ganglia-thalamic loop

**Figure 5.**
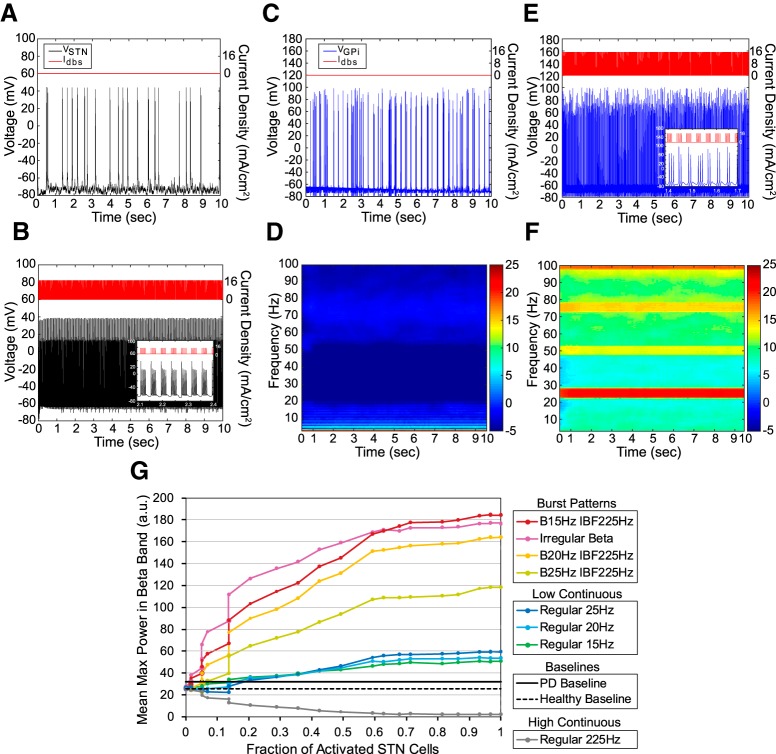
Effect of stimulation patterns on neuronal activity in a biophysically-based computational model of the basal ganglia. ***A***, Model STN neuron voltage output with no applied STN input. ***B***, Model STN neuron voltage output with pattern B25 IBF225 applied to STN and 100% STN cell activation (*n* = 59 cells). ***C***, Model GPi neuron voltage output with no applied STN input. ***D***, Averaged spectrogram of GPi neuron voltage output with no applied STN input (*n* = 59 cells). ***E***, Model GPi neuron voltage output with pattern B25 IBF225 applied to STN and 100% STN neuron activation. Inset, Burst activity in GPi neuron in response to burst patterned STN input. ***F***, Averaged spectrogram of GPi neuron voltage output with pattern B25 IBF225 applied to STN and 100% STN neuron activation (*n* = 59 cells). ***G***, Mean maximum power in the β band as a function of fraction of STN neurons activated and stimulation pattern. Dashed black line (“healthy baseline”) and solid black line (“PD baseline”) indicate baseline GPi β band power with no applied STN stimulation when model is run in either healthy or parkinsonian states, respectively. Two-way ANOVA performed on log-transformed data revealed a significant effect of pattern (*p* < 0.0001), log(fraction of activated STN cells; *p* < 0.0001), and a significant interaction term (*p* < 0.0001). *Post hoc* Tukey’s test found a significant difference between the increase in log(mean max β) caused by each bursting pattern, both regular and irregular, in the model as compared to the increase caused by each continuous low**-**frequency patterns (*p* < 0.0001 for all comparisons). Additionally, *post hoc* Tukey’s test found a significant difference between regular 225 Hz and Healthy Baseline (*p* < 0.0001) and between regular 225 Hz and PD baseline (*p* < 0.0001). Error bars indicate mean ± SD (*n* = 10 simulations).

The amount of β band power in the “healthy” model cortico-basal ganglia-thalamic loop was affected by stimulation pattern and the fraction of total cells activated with a significant interaction between pattern and fraction of cells activated (Fig. 5*G*); statistics are reported in Figure 5 and Table 2. While some BFP was present in the model network in the healthy state (Fig. 5*G*), β bursting rhythms were not present in the STN or GPi cell populations (Fig. 5*A*,*C*,*D*). When β-patterned stimulation was applied to model STN neurons, β band rhythms were seen in model STN and GPi neurons (Fig. 5*B*,*E*,*F*). *Post hoc* testing revealed that while continuous low-frequency stimulation paradigms at β band frequencies did increase averaged peak β band power, irregular and regular β stimulation paradigms increased β band power beyond that generated by continuous low-frequency stimulation (Fig. 5*G*). Also, continuous high-frequency stimulation suppressed power in the β band with increasing STN neuron activation as compared to the healthy model baseline and the “PD” model baseline (Fig. 5*G*). After confirming generation of BFP in the computational model, these same patterns were applied to healthy rats to quantify any resulting deteriorations in motor performance as well as to document an increase in BFP in SNr unit activity.

**Table 2.** Two-way ANOVA of the effects of β-patterned stimulation and fraction of total cells activated on the amount of β band power in the healthy biophysical circuit model of the cortico-basal ganglia-thalamic loop

Parameter	*p* value	*F*	df
Stimulation pattern	<0.0001	1118.4	9,34.7
Log(fraction of total cells activated)	<0.0001	729.3	1,2.51
Interaction term	<0.0001	314.0	9,9.73

### Effects of β-patterned STN stimulation on behavior in healthy rats

We administered five widely used motor tasks to maximize our ability to detect induction of bradykinesia/akinesia due to β-patterned stimulation. Although most of these tasks were sensitive to differences in performance between intact and 6-OHDA-lesioned rats, we did not detect stimulation-induced deteriorations in motor performance in healthy rats.

### Bar test

**Figure 6.**
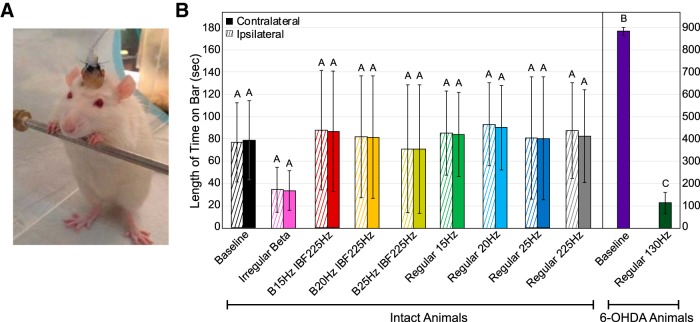
Effect of STN stimulation patterns on performance in bar test. “Contralateral” refers to the side of the body contralateral to the brain hemisphere to which stimulation was applied. Error bars indicate mean ± SE. Patterns not sharing the same letter are significantly different from each other (*p* < 0.05). ***A***, Rat was placed in an upright position with its forepaws resting on a bar 10 cm above the ground. ***B***, Length of time on bar as a function of stimulation pattern, lesion state, and forepaw. Two-way RMANOVA performed on healthy (intact) animal data were not significant in length of time on bar for pattern (*p* = 0.92), paw (*p* = 0.34), or pattern × paw (*p* = 0.74). See Table 1 for *n* for each pattern. A statistically significant difference in length of time on bar between baseline 6-OHDA**-**treated animal (*n* = 9) contralateral paw performance and intact animal (*n* = 7) baseline contralateral paw performance was found (*p* < 0.0001, Wilcoxon rank sum test). A statistically significant difference in length of time on bar between baseline 6-OHDA**-**treated animal performance (*n* = 9) performance and performance of 6-OHDA**-**treated animals with 130**-**Hz stimulation was found (*p* < 0.0001, Wilcoxon rank sum test).

Forelimb akinesia as assessed by length of time on the bar was unaffected by β-patterned stimulation (Table 3; Fig. 6). Use of the bar test detected a statistically significant worsening of forelimb akinesia after treatment with 6-OHDA that was improved by 130-Hz STN stimulation (Fig. 6*B*). However, we detected no differences in performance between β-patterned, continuous low-frequency, or continuous high-frequency paradigms in intact animals as compared to the no stimulation condition (Fig. 6*B*). There also was no impact of paw on the results nor a significant interaction between pattern and paw (Fig. 6*B*).

**Table 3.** Bar test: results of statistical analyses for effects on length of time on bar

Parameter	*n*/group	Test statistic	df	*p* value
Intact animals				
	Two-way RMANOVA (*F*)			
Stimulation pattern	See Table 1	0.40	8,36.1	0.92
Paw		1.06	1,6.9	0.34
Interaction term		0.64	8,38.2	0.74
6-OHDA-lesioned animals				
	Wilcoxon rank sum (χ^2^)			
Effect of 6-OHDA toxin	9 vs 7	15.8	1	<0.0001
Effect of 130-Hz stim after lesion	9/group	16.5	1	<0.0001

### Open field test

**Figure 7.**
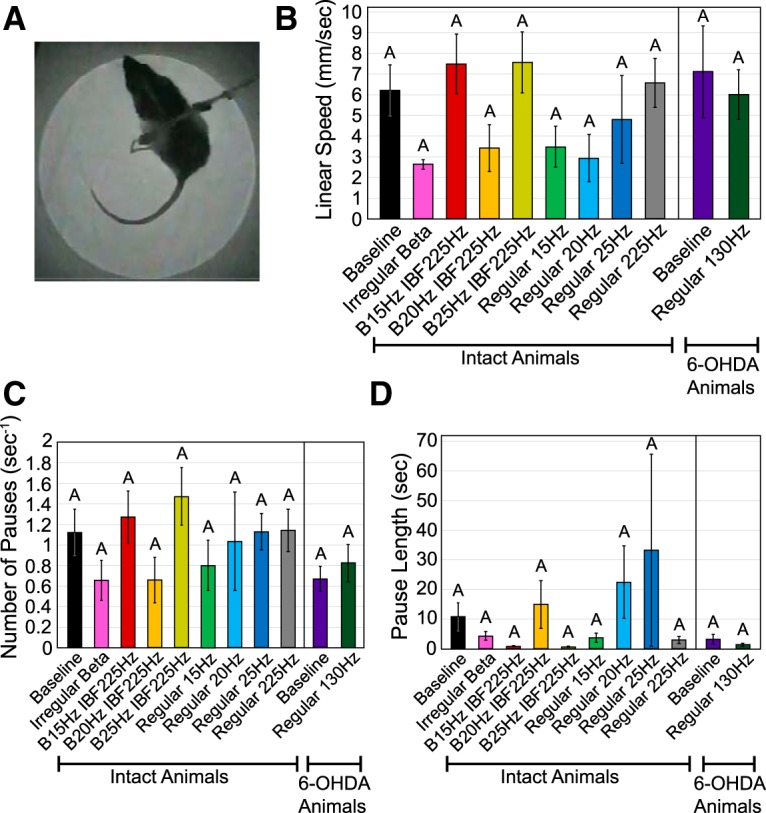
Effect of STN stimulation patterns on performance in open field test. Error bars indicate mean ± SE. Patterns not sharing the same letter are significantly different from each other (*p* < 0.05). ***A***, Rat was placed in a dark cylindrical chamber and its movements were video recorded. ***B***, Linear speed as a function of stimulation pattern and lesion state. One-way RMANOVA performed on intact animal data found no significant effect of pattern (*p* = 0.15). See Table 1 for *n* for each pattern. An unpaired *t* test performed on 6-OHDA**-**treated animal (*n* = 5) baseline performance versus intact animal (*n* = 9) baseline performance found no significant effect of lesion state (*p* = 0.72). A paired *t* test performed on 6-OHDA**-**treated animal data (*n* = 5) found no significant effect of 130-Hz stimulation (*p* = 0.54). ***C***, Number of pauses per second as a function of stimulation pattern and lesion state. One-way RMANOVA performed on intact animal data found no significant effect of pattern (*p* = 0.16). See Table 1 for *n* for each pattern. An unpaired *t* test performed on 6-OHDA**-**treated animal (*n* = 5) baseline performance versus intact animal (*n* = 9) baseline performance found no significant effect of lesion state (*p* = 0.11). A paired *t* test performed on 6-OHDA**-**treated animal data (*n* = 5) found no significant effect of 130-Hz stimulation (*p* = 0.22). ***D***, Pause length as a function of stimulation pattern and lesion state. One-way RMANOVA performed on intact animal data found no significant effect of pattern (*p* = 0.44). See Table 1 for *n* for each pattern. An unpaired *t* test performed on 6-OHDA**-**treated animal (*n* = 5) baseline performance versus intact animal (*n* = 9) baseline performance found no significant effect of lesion state (*p* = 0.17). A paired *t* test performed on 6-OHDA**-**treated animal data (*n* = 5) found no significant effect of 130-Hz stimulation (*p* = 0.30).

The open field test did not detect differences in locomotor activity across stimulation paradigms (Table 4; Fig. 7). β-Patterned, continuous low-frequency, and continuous high-frequency paradigms did not evoke discernable differences in linear speed (Fig. 7*B*), number of pauses per second (Fig. 7*C*), or pause length (Fig. 7*D*) in intact animals as compared to the no stimulation condition. However, 6-OHDA**-**treated animal performance also did not differ significantly from intact animal performance either at baseline or during 130-Hz STN stimulation (Fig. 7*B–D*). The average linear speed during the no stimulation condition was only 7 mm/s (Fig. 7*B*) implying that animals were not exploring the arena during the experiment, thus making it difficult to detect differences in activity across patterns or lesion states.

**Table 4.** Open field test: results of statistical analyses for effects on locomotor activity

Metric	Parameter	*n*/group	Test statistic	df	*p* value
Intact animals					
		One-way RMANOVA (*F*)			
Linear speed	Stimulation pattern	See Table 1	1.60	8,42.7	0.15
Pauses per second	Stimulation pattern		1.58	8,42.6	0.16
Pause length	Stimulation pattern		1.01	8,45.2	0.44
6-OHDA-lesioned animals					
		Unpaired *t* test (*t*)			
Linear speed	6-OHDA toxin effect	5 vs 9	0.36	6.5	0.73
Pauses per second	6-OHDA toxin effect		–1.75	11.4	0.11
Pause length	6-OHDA toxin effect		–1.49	9.9	0.17
		Paired *t* test (*t*)			
Linear speed	130-Hz stim after lesion	5/group	0.67	4	0.54
Pauses per second	130-Hz stim after lesion		–1.47	4	0.22
Pause length	130-Hz stim after lesion		1.19	4	0.30

### Adjusting steps test

**Figure 8.**
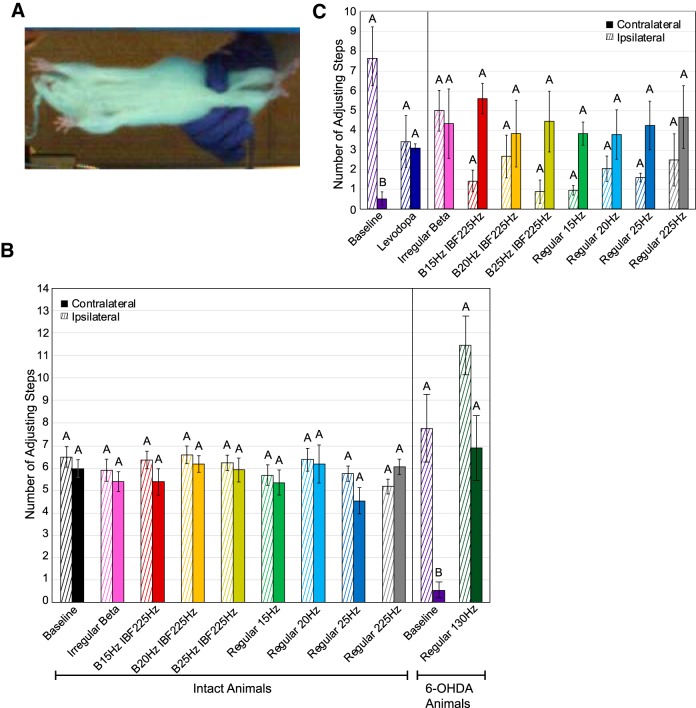
Effect of STN stimulation patterns on performance in adjusting steps test. “Contralateral” refers to the side of the body contralateral to the brain hemisphere to which stimulation was applied. Error bars indicate mean ± SE. Patterns not sharing the same letter are significantly different from each other (*p* < 0.05). ***A***, The rat was suspended such that its forepaws bore its weight as it was dragged backwards along a clear glass surface. Adjusting steps were video recorded from below. ***B***, Number of forepaw adjusting steps made as a function of stimulation pattern, lesion state, and forepaw. Two-way RMANOVA performed on intact animal data were not significant for pattern (*p* = 0.48), paw (*p* = 0.28), or pattern × paw (*p* = 0.10). See Table 1 for *n* for each pattern. Two-way RMANOVA performed between intact animal data (*n* = 8) and baseline 6-OHDA**-**treated animal data (*n* = 6) found significant effects of lesion state (*p* = 0.0498), paw (*p* = 0.039), and lesion state × paw (*p* = 0.0007). *Post hoc* Tukey’s test on lesion state × paw interaction found significant differences between 6-OHDA**-**treated and intact animal contralateral paw performance (*p* = 0.0041) and between 6-OHDA**-**treated animal contralateral and ipsilateral paw performance (*p* = 0.0003). Two-way RMANOVA performed on 6-OHDA**-**treated animal data found a significant effect of pattern (*p* = 0.019), a significant effect of paw (*p* = 0.024), but no significant interaction term. ***C***, Number of forepaw adjusting steps made by levodopa-treated 6-OHDA rats as a function of stimulation pattern, drug state, and forepaw. An unpaired *t* test performed on contralateral paw performance with (*n* = 3) and without (*n* = 6) levodopa and without applied stimulation found a significant effect of drug state (*p* = 0.0001). Two-way RMANOVA performed on levodopa-treated 6-OHDA rats was not significant for pattern (*p* = 0.31), paw (*p* = 0.10), or pattern × paw (*p* = 0.13). See Table 1 for *n* for each pattern.

Forelimb akinesia as assessed by the adjusting steps test was not appreciably different with β-patterned, continuous low-frequency, or continuous high-frequency stimulation in intact animals as compared to the no stimulation condition (Table 5; Fig. 8). There was no impact of paw on the intact animal data nor a significant interaction between pattern and paw (Fig. 8*B*). Treatment with 6-OHDA evoked a significant worsening of forelimb akinesia as compared to intact animals specifically in the contralateral paw (Fig. 8*B*). Additionally, the interaction between lesion state and paw was significant with *post hoc* Tukey’s tests demonstrating significant differences in contralateral paw performance between 6-OHDA**-**treated and intact animals (*p* = 0.0041) and between contralateral and ipsilateral paw performance in 6-OHDA**-**treated animals (*p* = 0.0003). Stimulation with regular 130 Hz ameliorated the dysfunction of the contralateral paw in 6-OHDA**-**treated animals (Fig. 8*B*).

**Table 5.** Adjusting steps test: results of statistical analyses for effects on number of steps

Parameter	*n*/group	Test statistic	df	*p* value
Intact animals				
	Two-way RMANOVA (*F*)			
Stimulation pattern	See Table 1	0.96	8,35.6	0.48
Paw		1.38	1,7.72	0.28
Interaction term		1.83	8,36.9	0.10
6-OHDA-lesioned animals				
	Two-way RMANOVA (*F*)			
6-OHDA toxin	8 vs 6	4.91	1,10.5	0.0498
Paw		26.5	1,1.93	0.039
Interaction term		22.9	1,10.1	0.0007
	Two-way RMANOVA (*F*)			
130-Hz stimulation	8 vs 6	40.6	1,2.21	0.019
Paw		17.0	1,3.14	0.024
Interaction term		1.06	1,2.74	0.38
	Unpaired *t* test (*t*)			
Levodopa effect on contralateral paw	6 vs 3	–7.78	6.63	0.0001
6-OHDA-lesioned animals + levodopa				
	Two-way RMANOVA (*F*)			
Stimulation pattern	See Table 1	1.31	8,16	0.31
Paw		8.37	1,2	0.10
Interaction term		1.89	8,16	0.13

### Forelimb use asymmetry test

**Figure 9.**
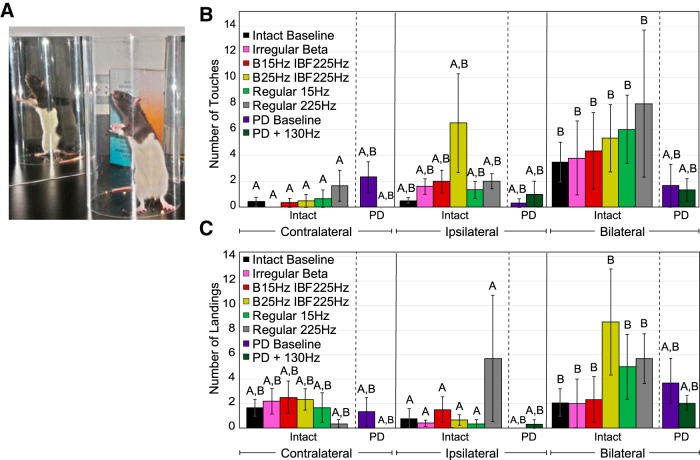
Effect of STN stimulation patterns on performance in the forelimb use asymmetry test. “Contralateral” refers to the side of the body contralateral to the brain hemisphere to which stimulation was applied. Error bars indicate mean ± SE. Patterns not sharing the same letter are significantly different from each other (*p* < 0.05). ***A***, Rat was placed in a clear cylinder and vertical exploration of the cylinder was video recorded. ***B***, Preferred forelimb use in vertical exploration as a function of stimulation pattern and lesion state. Two-way RMANOVA performed on intact animal data revealed a significant effect of paw (*p* = 0.026), but no significant effect of pattern (*p* = 0.55) or pattern × paw (*p* = 0.72). See Table 1 for *n* for each pattern. *Post hoc* Tukey’s test on factor paw found bilateral touches during vertical exploration to be significantly greater than contralateral paw touches across all stimulation patterns in intact animals (*n* = 8, *p* = 0.021). Two-way RMANOVA performed on intact (*n* = 8) versus 6-OHDA**-**treated (*n* = 3) animal baseline performance found no significant effect of paw (*p* = 0.10), lesion state (*p* = 0.98), or paw × lesion state (*p* = 0.34). Two-way RMANOVA performed on 6-OHDA**-**treated (*n* = 3) animal data found no significant effect of paw (*p* = 0.53), pattern (*p* = 0.42), or paw × pattern (*p* = 0.44) with 130-Hz stimulation. ***C***, Preferred forelimb use in landing as a function of stimulation pattern and lesion state. Two-way RMANOVA performed on intact animal data found a significant effect of paw (*p* = 0.021), but no significant effect of pattern (*p* = 0.36) or pattern × paw (*p* = 0.32). See Table 1 for *n* for each pattern. *Post hoc* Tukey’s test on factor paw found bilateral forelimb use during landings to be significantly greater than ipsilateral forelimb use in landings across all stimulation patterns in intact animals (*p* = 0.032). Two-way RMANOVA performed on intact (*n* = 8) versus 6-OHDA**-**treated (*n* = 3) animal baseline performance found no significant effect of paw (*p* = 0.06), lesion state (*p* = 0.87), or paw × lesion state (*p* = 0.45). Two-way RMANOVA performed on 6-OHDA**-**treated (*n* = 3) animal data found no significant effect of paw (*p* = 0.07), pattern (*p* = 0.42), or paw × pattern (*p* = 0.44) with 130-Hz stimulation.

No differences in forelimb akinesia as quantified by the cylinder test were detected with β-patterned, continuous low-frequency, or continuous high-frequency stimulation in intact animals as compared to the no stimulation condition (Table 6; Fig. 9), but these results are limited in a manner similar to those of the open field test. There was a significant effect of paw in both vertical exploration (Fig. 9*B*) and landings (Fig. 9*C*). Intact animals preferred bilateral forelimb use during vertical exploration to contralateral forelimb use (*p* = 0.026, Tukey’s HSD), and this was unaffected by stimulation pattern. Similarly, when landing from a rear, intact animals preferred a balanced landing to ipsilateral forelimb (*p* = 0.032, Tukey’s HSD) use across stimulation conditions. Interactions between pattern and paw were not significant for either vertical exploration or landings. 6-OHDA**-**treated animal performance did not differ significantly from intact animal performance, and performance in 6-OHDA**-**treated animals was not improved with 130-Hz stimulation (vertical exploration: Fig. 9*B*; landings: Fig. 9*C*). There was no effect of paw or a significant interaction term in either case. All lesions were confirmed *in vivo* through use of the methamphetamine induced circling test. However, intact animal baseline performance was limited (Fig. 9*B*,*C*), and animals lost interest in exploring the cylinder on repeated exposures. Thus, differences between intact and 6-OHDA**-**treated animal performance were difficult to detect.

**Table 6.** Forelimb use asymmetry test: results of statistical analyses for effects on forelimb akinesia

Metric	Parameter	*n*/group	Test statistic	df	*p* value
Intact animals					
		Two-way RMANOVA (*F*)			
Number of touches (vertical)	Stimulation pattern	See Table 1	0.84	5,11.1	0.55
	Paw		5.09	2,11.5	0.026
	Interaction		0.69	10,21.5	0.72
Number of landings	Stimulation pattern	See Table 1	1.13	5,63.8	0.36
	Paw		4.21	2,95.6	0.021
	Interaction		1.19	10,136	0.32
6-OHDA-lesioned animals					
		Two-way RMANOVA (*F*)			
Number of touches (vertical)	6-OHDA toxin effect	8 vs 3	0.0004	1,0.002	0.98
	Paw		2.52	2,23.83	0.10
	Interaction		1.14	2,10.72	0.34
Number of touches (vertical)	130-Hz stim after lesion	3/group	1.00	1,2	0.42
	Paw		0.74	2,4	0.53
	Interaction		1.00	2,4	0.44
		Two-way RMANOVA (*F*)			
Number of landings	6-OHDA toxin effect	8 vs 3	0.027	1,6	0.87
	Paw		3.65	2,12	0.06
	Interaction		0.85	2,12	0.45
Number of landings	130-Hz stim after lesion	3/group	1.00	1,2	0.42
	Paw		5.45	2,4	0.07
	Interaction		1.00	2,4	0.44

### Skilled forelimb reaching test

**Figure 10.**
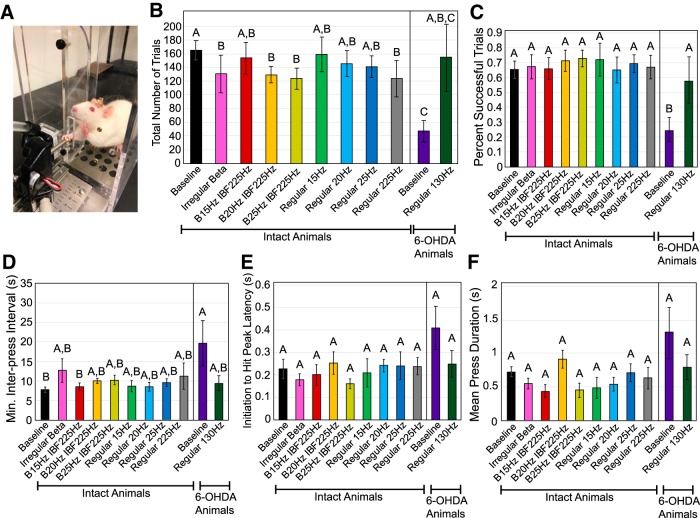
Effect of STN stimulation pattern on performance in the skilled forelimb reaching test. Error bars indicate mean ± SE. Patterns not sharing the same letter are significantly different from each other (*p* < 0.05). ***A***, Rat was placed in a clear chamber with a slot to allow it to grasp and depress a lever using only the forelimb contralateral to stimulation or lesioned hemisphere. ***B***, Total number of trials per experimental session as a function of stimulation pattern and lesion state. One-way RMANOVA performed on intact animal data found a significant effect of pattern (*p* = 0.0024). See Table 1 for *n* for each pattern. *Post hoc* Tukey’s test on factor pattern found significant differences between baseline and the following patterns: irregular β (*p* = 0.044), B20IBF225 (*p* = 0.035), B25IBF225 (*p* = 0.021), and regular 225 Hz (*p* = 0.0025). One-way unpaired *t* test performed on 6-OHDA**-**treated (*n* = 4) animal baseline performance versus intact animal (*n* = 8) performance found a significant effect of lesion state (*p* = 0.0003). One-way matched pairs *t* test performed on 6-OHDA**-**treated animal data with (*n* = 3) and without (*n* = 3) 130-Hz stimulation found a near significant effect of stimulation (*p* = 0.11). ***C***, Percentage of trials resulting in pellet dispensation as a function of stimulation pattern and lesion state. One-way RMANOVA performed on intact animal data found no significant effect of pattern (*p* = 0.30). See Table 1 for *n* for each pattern. One-way unpaired *t* test performed on 6-OHDA**-**treated (*n =* 4) animal baseline performance versus intact animal (*n* = 8) performance found a significant effect of lesion state (*p* = 0.0025). One-way matched pairs *t* test performed on 6-OHDA**-**treated animal data with (*n* = 3) and without (*n* = 3) 130-Hz stimulation found a significant effect of 130-Hz stimulation (*p* = 0.017). ***D***, Minimum interpress interval as a function of stimulation pattern and lesion state. One-way RMANOVA performed on intact animal data found no significant effect of pattern (*p* = 0.21). See Table 1 for *n* for each pattern. One-way unpaired *t* test performed on 6-OHDA**-**treated (*n* = 4) animal baseline performance versus intact animal (*n* = 8) performance found a significant effect of lesion state (*p* = 0.041). One-way matched pairs *t* test performed on 6-OHDA**-**treated animal data with (*n* = 3) and without (*n* = 3) 130-Hz stimulation found no significant effect of 130-Hz stimulation (*p* = 0.24). ***E***, Initiation to hit peak latency as a function of stimulation pattern and lesion state. One-way RMANOVA performed on intact animal data found no significant effect of pattern (*p* = 0.44). See Table 1 for *n* for each pattern. One-way unpaired *t* test performed on 6-OHDA**-**treated (*n* = 4) animal baseline performance versus intact animal (*n* = 8) performance found a near significant effect of lesion state (*p* = 0.07). One-way matched pairs *t* test performed on 6-OHDA**-**treated animal data with (*n* = 3) and without (*n* = 3) 130-Hz stimulation found no significant effect of 130-Hz stimulation (*p* = 0.17). ***F***, Mean press duration as a function of stimulation pattern and lesion state. One-way RMANOVA performed on intact animal data found a significant effect of pattern (*p* = 0.037), but *post hoc* Tukey’s test found no significant differences among patterns. See Table 1 for *n* for each pattern. One-way unpaired *t* test performed on 6-OHDA**-**treated (*n* = 4) animal baseline performance versus intact animal (*n* = 8) performance found a near significant effect of lesion state (*p* = 0.15). One-way matched pairs *t* test performed on 6-OHDA**-**treated animal data with (*n* = 3) and without (*n* = 3) 130-Hz stimulation found no significant effect of 130-Hz stimulation (*p* = 0.20).

No worsening of forelimb bradykinesia as assessed by the lever press was detected with β-patterned, continuous low-frequency, or continuous high-frequency stimulation (Table 7; Fig. 10). Stimulation in intact animals caused a statistically significant worsening from intact baseline performance only for total trials attempted (Fig. 10*B*). *Post hoc* Tukey’s tests showed that patterns irregular β (*p* = 0.044), B20 Hz IBF225 Hz (*p* = 0.035), B25 Hz IBF225 Hz (*p* = 0.021), and regular 225 Hz (*p* = 0.0025) all caused a decrease in total trials attempted from the no stimulation baseline condition. However, given that the overall success rate at triggering pellet release and quantitative metrics of lever push and release dynamics were unaffected by stimulation pattern, this finding is likely not physiologically relevant. A statistically significant change in mean press duration with stimulation in intact animals also was observed (Fig. 10*F*), but *post hoc* Tukey’s testing found no significant differences among patterns indicating that this finding also is likely not physiologically relevant.

**Table 7.** Skilled forelimb reaching test: results of statistical analyses for effects on forelimb bradykinesia

Metric	Parameter	*n*/group	Test statistic	df	*p* value
Intact animals					
		One-way RMANOVA (*F*)			
Total trials attempted	Stim pattern	See Table 1	3.77	8,38.5	0.0024
% Successful trials	Stim pattern		1.28	8,23.5	0.30
Minimum interpress interval	Stim pattern		1.43	8,43.6	0.21
Initiation to hit peak latency	Stim pattern		1.06	8,43.6	0.44
Mean press duration	Stim pattern		2.62	8,20.6	0.037
6-OHDA-lesioned animals					
		Unpaired *t* test (*t*)			
Total trials attempted	6-OHDA toxin	4 vs 8	–5.63	7.46	0.0003
% Successful trials	6-OHDA toxin		–4.13	6.61	0.0025
Minimum interpress interval	6-OHDA toxin		2.40	3.57	0.041
Initiation to hit peak latency	6-OHDA toxin		1.80	4.35	0.07
Mean press duration	6-OHDA toxin		1.23	3.58	0.15
		One-way matched pairs *t* test (*t*)			
Total trials attempted	130-Hz stim	3/group	1.76	2	0.11
% Successful trials	130-Hz stim		5.31	2	0.017
Minimum interpress interval	130-Hz stim		–0.86	2	0.24
Initiation to hit peak latency	130-Hz stim		–1.24	2	0.17
Mean press duration	130-Hz stim		–1.05	2	0.20
		One-way matched pairs *t* test (*t*)			
Total trials attempted	Levodopa	3/group	4.20	2	0.026
% Successful trials	Levodopa		5.51	2	0.016
Minimum interpress interval	Levodopa		–0.84	2	0.24
Initiation to hit peak latency	Levodopa		–1.32	2	0.16
Mean press duration	Levodopa		–1.71	2	0.11
6-OHDA-lesioned animals + Levodopa					
		One-way RMANOVA (*F*)			
Total trials attempted	Stim pattern	See Table 1	0.38	8,16	0.92
% Successful trials	Stim pattern		0.84	8,16	0.58
Minimum interpress interval	Stim pattern		1.09	8,16	0.42
Initiation to hit peak latency	Stim pattern		1.19	8,16	0.36
Mean press duration	Stim pattern		1.05	8,16	0.44

The skilled forelimb reaching test did detect significant differences between performance of intact and 6-OHDA**-**treated animals (Table 7; Fig. 10*B–F*). Treatment with 6-OHDA caused a worsening of total trials attempted (Fig. 10*B*); success rate (Fig. 10*C*); and minimum interpress interval (Fig. 10*D*). These results indicate that 6-OHDA-lesioned rats attempted fewer trials, were less successful at triggering pellet release when trials were attempted, and waited longer to initiate subsequent trials than intact rats. Additionally, a near significant effect was found for initiation to hit peak latency (Fig. 10*E*) and mean press duration (Fig. 10*F*). These results indicate that 6-OHDA-lesioned rats trended toward an increase in time between the first and second push in a given trial as indicated by the effect of lesion state on initiation to hit peak latency. 6-OHDA-lesioned rats also trended toward taking longer to release the lever after each push as quantified by the mean press duration metric.

**Figure 11.**
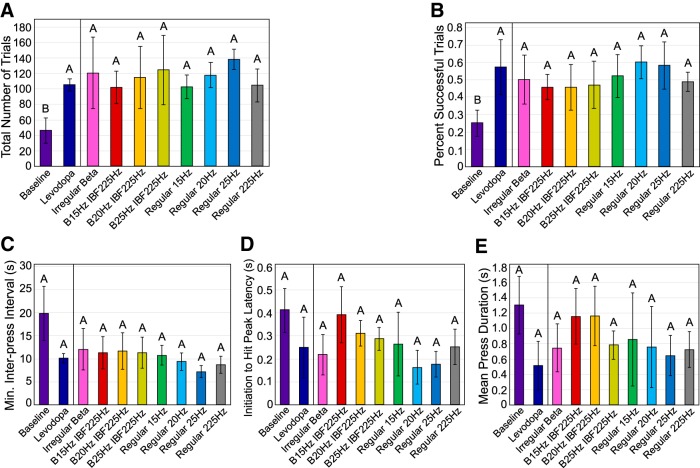
Effect of STN stimulation pattern on performance by levodopa-treated 6-OHDA rats in the skilled forelimb reaching test. Error bars indicate mean ± SE. Patterns not sharing the same letter are significantly different from each other (*p* < 0.05). See Table 1 for *n* for each pattern. ***A***, Total number of trials per experimental session as a function of stimulation pattern and drug condition. One-way matched pairs *t* test performed on 6-OHDA**-**treated animal baseline performance without stimulation or levodopa (*n* = 3) versus with levodopa (*n* = 3) found a significant effect of drug (*p* = 0.026). One-way RMANOVA performed levodopa-treated 6-OHDA animal performance found no significant effect of stimulation pattern (*p* = 0.92). ***B***, Percentage of trials resulting in pellet dispensation as a function of stimulation pattern and drug condition. One-way matched pairs *t* test performed on 6-OHDA**-**treated animal baseline performance without stimulation or levodopa (*n* = 3) versus with levodopa (*n* = 3) found a significant effect of drug (*p* = 0.016). One-way RMANOVA performed on levodopa-treated 6-OHDA animal performance found no significant effect of stimulation pattern (*p* = 0.58). ***C***, Minimum interpress interval as a function of stimulation pattern and drug condition. One-way matched pairs *t* test performed on 6-OHDA**-**treated animal baseline performance without stimulation or levodopa (*n* = 3) versus with levodopa (*n* = 3) found no significant effect of drug (*p* = 0.24). One-way RMANOVA performed on levodopa-treated 6-OHDA animal performance found no significant effect of stimulation pattern (*p* = 0.42). ***D***, Initiation to hit peak latency as a function of stimulation pattern and drug condition. One-way matched pairs *t* test performed on 6-OHDA**-**treated animal baseline performance without stimulation or levodopa (*n* = 3) versus with levodopa (*n* = 3) found no significant effect of drug (*p* = 0.16). One-way RMANOVA performed on levodopa-treated 6-OHDA animal performance found no significant effect of stimulation pattern (*p* = 0.36). ***E***, Mean press duration as a function of stimulation pattern and drug condition. One-way matched pairs *t* test performed on 6-OHDA**-**treated animal baseline performance without stimulation or levodopa (*n* = 3) versus with levodopa (*n* = 3) found no significant effect of drug (*p* = 0.15). One-way RMANOVA performed on levodopa-treated 6-OHDA animal performance found no significant effect of stimulation pattern (*p* = 0.44).

A 130-Hz stimulation applied to 6-OHDA-lesioned rats improved the fraction of trials that produced a pellet (Table 7; Fig. 10*C*). Figure 10 also demonstrates a near significant effect of 130-Hz stimulation on total trials attempted (Fig. 10*B*) indicating a trend toward an increase in total trials attempted when 130-Hz stimulation was applied. Trends toward a rescue of performance on interpress interval (Fig. 10*D*), initiation to hit peak latency (Fig. 10*E*), and mean level press duration (Fig. 10*F*) were seen with 130-Hz stimulation. Additionally, the trends seen with 130-Hz stimulation were similar to the improvements in 6-OHDA-lesioned animal performance seen with levodopa administration, which were less variable across rats and were statistically significant (Fig. 11). In particular, injection of levodopa increased total number of trials attempted and success rate.

### Effects of β-patterned stimulation on behavior in 6-OHDA-lesioned rats

We applied patterns of STN stimulation to 6-OHDA-lesioned animals pre-treated with levodopa to control and increase BFP and assess whether the neural substrate changes inherent to the parkinsonian brain would amplify the effect of β-patterned paradigms on motor function.

### Adjusting steps test

Application of β-patterned stimulation paradigms to levodopa-treated 6-OHDA-lesioned rats did not cause a detectable deterioration in motor performance in the paw contralateral to STN stimulation (Table 5; Fig. 8). Additionally, there was no detectable effect of paw or an interaction of paw × stimulation pattern. Treatment with levodopa improved use of the contralateral paw in 6-OHDA**-**treated rats (Fig. 8*C*), but paw performance remained consistently high across β-patterned, continuous low-frequency, and continuous high-frequency paradigms.

### Skilled forelimb reaching test

Injection of levodopa significantly improved the total number of trials attempted (Fig. 11*A*) and the rate of successful pellet release (Fig. 11*B*), and there were trends toward decreasing the intertrial interval (Fig. 11*C*), the initiation to hit peak latency (Fig. 11*D*), and the mean press duration (Table 7; Fig. 11*E*). However, β-patterned stimulation paradigms did not effect any detectable changes in performance metrics quantified by the lever press task as compared to levodopa alone (Fig. 11). The effect of stimulation patterns did not appear different from the effect of levodopa alone for the total number of trials (Fig. 11*A*), success rate (Fig. 11*B*), intertrial interval (Fig. 11*C*), initiation to hit peak latency (Fig. 11*D*), and mean press duration (Fig. 11*E*) metrics. These data indicate that the stimulation patterns did not induce detectable symptoms of bradykinesia/akinesia in our levodopa-treated 6-OHDA-lesioned rat model.

### Pre-6-OHDA single unit recordings

**Figure 12.**
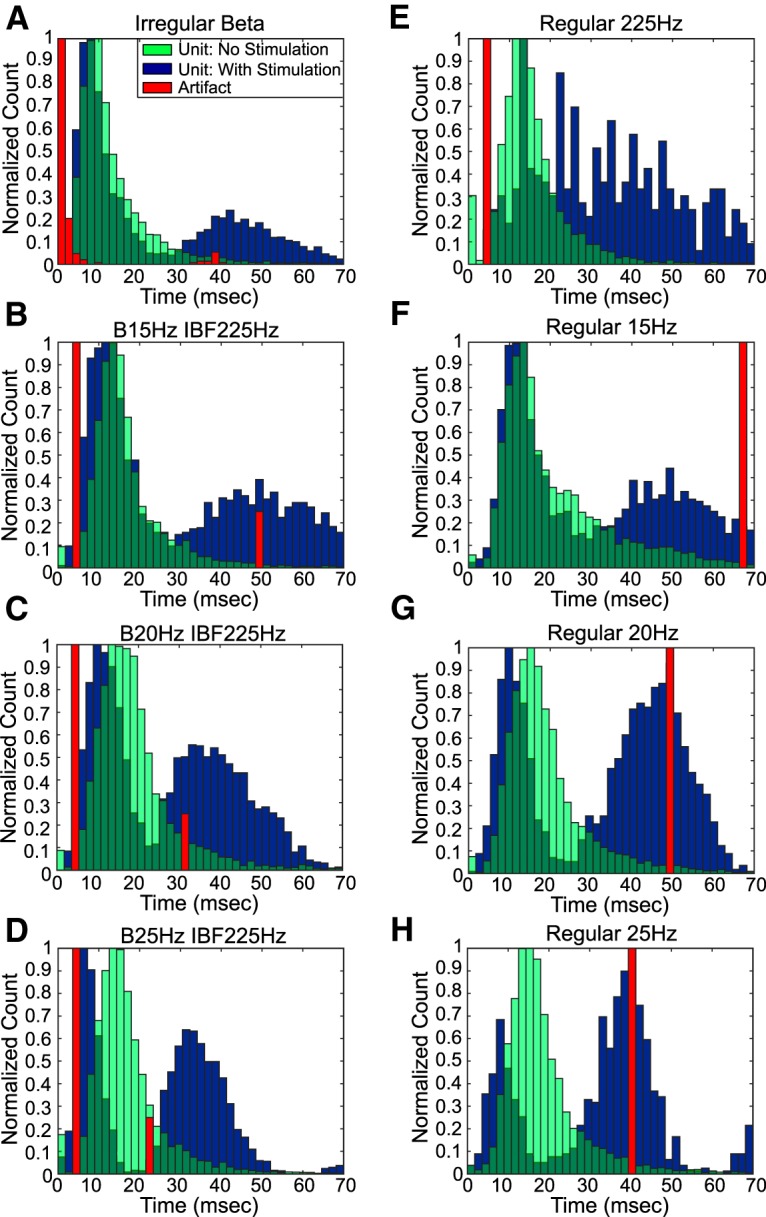
Effect of STN stimulation patterns on single unit ISI histograms. ***A****–****H***, Normalized histograms for a representative SNr unit representing the pre-stimulation and stimulation periods. Stimulation causes a shift in the unit ISI histogram to mimic the artifact IPI histogram.

**Figure 13.**
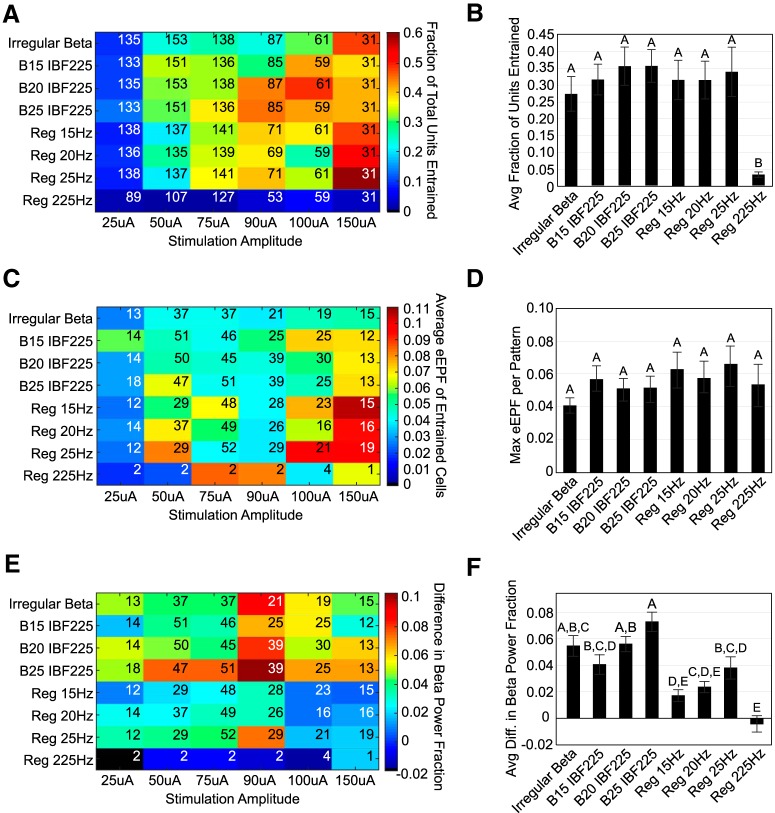
Effect of STN stimulation patterns on eEPF. Patterns not connected by the same letter are significantly different from each other (*p* < 0.05). Error bars indicate mean ± SE. ***A***, Fraction of total SNr units entrained as a function of stimulation pattern and amplitude. Inset numbers refer to total number of units assessed (*n*) per stimulation pattern and amplitude setting. Two-way ANOVA performed on entrainment fractions found a significant effect of pattern (*p* < 0.0001), amplitude (*p* < 0.0001), and pattern × amplitude (*p* = 0.030). ***B***, *Post hoc* Tukey’s test results for factor pattern across amplitude for entrainment fraction ANOVA. *Post hoc* Tukey’s testing showed a significant difference between regular 225 Hz and all other patterns (*p* < 0.0001 for all comparisons); all other patterns were equivocal in degree of entrainment. ***C***, Average eEPF of entrained SNr units as a function of stimulation pattern and amplitude. Inset numbers refer to total number of entrained units (*n*) included in the calculation. Two-way ANOVA found a significant effect of amplitude (*p* < 0.0001), but no significant effect of pattern (*p* = 0.43) or pattern × amplitude (*p* = 0.69). ***D***, *Post hoc* Tukey’s test results for factor pattern across amplitude for average eEPF value. No significant differences across pattern were found (*p* < 0.05). ***E***, Difference in β power fraction between stimulation and pre-stimulation power spectra as a function of stimulation pattern and amplitude. Inset numbers refer to total number of entrained units (*n*) included in the calculation. Two-way ANOVA found a significant effect of pattern (*p* < 0.0001), but no significant effect of amplitude (*p* = 0.54) or pattern × amplitude (*p* = 0.82). ***F***, *Post hoc* Tukey’s test results for factor pattern across amplitude for difference in β power ANOVA. *Post hoc* Tukey’s testing showed multiple significant differences between bursting patterns and continuous frequency patterns (*p* < 0.05).

As β-patterned stimulation did not cause a discernible worsening of motor performance in our behavioral tasks, we quantified SNr unit responses to STN stimulation to determine that we were indeed generating BFP with stimulation. The effect of STN stimulation on neural activity is displayed in Figure 12 for a representative SNr unit and summarized in Figure 13. STN stimulation caused changes in the ISI histograms from the pre-stimulation condition that mimicked the IPI histogram of the applied stimulation pattern. Figure 13*A* displays the fraction of total units assessed at each combination of stimulation pattern and amplitude. For a given pattern and amplitude, entrainment was defined as the presence of at least two consecutive bins in the normalized stimulation PSTH with counts above the z = 4 threshold. Entrainment fraction increased significantly with increasing amplitude (Table 8; Fig. 13*A*). A significant effect of pattern and a significant interaction term were also found (Table 8; Fig. 13*A*). Patterns generated equivocal degrees of entrainment with the exception of the continuous high-frequency pattern, which was significantly worse at entraining units than β-patterned or continuous low-frequency paradigms (*p* < 0.0001 for all comparisons, Tukey’s HSD; Fig. 13*B*).

**Table 8.** Effect of STN stimulation patterns on SNr unit entrainment: results of statistical analyses for effects on SNr units in intact, 6-OHDA lesioned, and 6-OHDA-lesioned + levodopa-treated animals

Metric	Parameter	*n*/group	Test statistic	df	*p* value
SNr units in intact animals (pre-6-OHDA)					
		Two-way ANOVA (*F*)			
Entrainment fraction	Amplitude	See Figure 13*A*, inset	100.1	1,0.44	<0.0001
	Stim pattern		15.2	7,0.47	<0.0001
	Interaction		2.61	7,0.08	0.03
Average eEPF	Amplitude	See Figure 13*C*, inset	25.8	1,0.008	<0.0001
	Stim pattern		1.03	7,0.002	0.43
	Interaction		0.68	7,0.001	0.69
Difference in BPF	Amplitude	See Figure 13*E*, inset	0.38	1,0.0001	0.54
	Stim pattern		11.9	7,0.024	<0.0001
	Interaction		0.50	7,0.001	0.82
SNr units in post-6-OHDA-lesioned animals					
		Two-way ANOVA (*F*)			
Entrainment fraction	Amplitude	See Figure 16*A*, inset	56.4	1,0.80	<0.0001
	Stim pattern		3.03	7,0.30	0.03
	Interaction		0.36	7,0.036	0.91
Average eEPF	Amplitude	See Figure 16*C*, inset	0.43	1,0.001	0.52
	Stim pattern		1.22	7,0.027	0.35
	Interaction		0.10	7,0.002	0.99
Difference in BPF	Amplitude	See Figure 16*E*, inset	6.63	1,0.0004	0.02
	Stim pattern		19.7	7,0.009	<0.0001
	Interaction		0.91	7,0.0004	0.53
SNr units in post-6-OHDA-lesioned animals + levodopa					
		Two-way ANOVA (*F*)			
Entrainment fraction	Drug state	See Figure 16*G*, inset	8.43	1,0.17	0.023
	Stim pattern		0.72	6,0.086	0.65
	Interaction		0.80	6,0.096	0.60
Average eEPF	Drug state	See Figure 16*H*, inset	0.17	1,5.6e-5	0.69
	Stim pattern		0.73	6,0.001	0.64
	Interaction		2.04	6,0.004	0.19
Difference in BPF	Drug state	See Figure 16*I*, inset	22.47	1,0.0004	0.0021
	Stim pattern		24.21	6,0.003	0.0002
	Interaction		2.07	6,0.0002	0.18

BPF = β power fraction.

The average eEPF of entrained units as a function of stimulation pattern and amplitude is depicted in Figure 13*C*. Increasing stimulation amplitude caused a statistically significant increase in eEPF (Table 8; Fig. 13*C*). The maximum eEPF values for each pattern ranged from 0.048 to 0.106 (Fig. 13*D*), indicating that 4.8–10.6% of spikes recorded during stimulation were generated by the corresponding stimulation pattern. However, no statistically significant differences in average eEPF were found across patterns, nor was a significant interaction term found (Table 8; Fig. 13*C*). For β-patterned paradigms at the highest amplitudes, the latency of peak entrainment occurred in a range of 3–7.2 ms following a stimulus pulse. Additional smaller peaks at later times were also observed but were variable. For continuous low-frequency stimulation at the highest amplitudes, two large peaks of entrainment occurred in latency ranges of 2.6–4.4 and 22.6–25.4 ms following a stimulus pulse. The continuous high-frequency control had a peak of entrainment at a latency of 2.6–4.4 ms.

The difference in BFP of the SNr units between the baseline and stimulation conditions as a function of stimulation pattern and amplitude are displayed in Figure 13*E*. There was a significant effect of pattern (Table 8; Fig. 13*E*) with a tendency toward burst-patterned paradigms causing greater increases in BFP in SNr units with stimulation as compared to the pre-stimulation baseline. In particular, *post hoc* Tukey’s testing showed that the B25 Hz IBF225 Hz pattern increased SNr unit BFP to a greater degree than the B15 IBF225 Hz (*p* = 0.04), regular 15 Hz (*p* < 0.0001), regular 20 Hz (*p* = 0.0004), regular 25 Hz (*p* = 0.02), and regular 225 Hz (*p* < 0.0001) patterns (Fig. 13*F*). The B20 IBF225 Hz and irregular β patterns also increased SNr unit BFP to a greater degree than the regular 15 Hz (B20 IBF225 Hz vs regular 15 Hz: *p* = 0.008; irregular β vs regular 15 Hz: *p* = 0.01) and regular 225 Hz (B20 IBF225 Hz vs regular 225 Hz: *p* < 0.0001; irregular β vs regular 225 Hz: *p* < 0.0001) patterns (Fig. 13*F*). Finally, the B20 IBF 225 Hz pattern increased SNr unit BFP to a greater degree than the regular 20 Hz (*p* = 0.04; Fig. 13*F*).

### Pre-6-OHDA ECoG recordings

**Figure 14.**
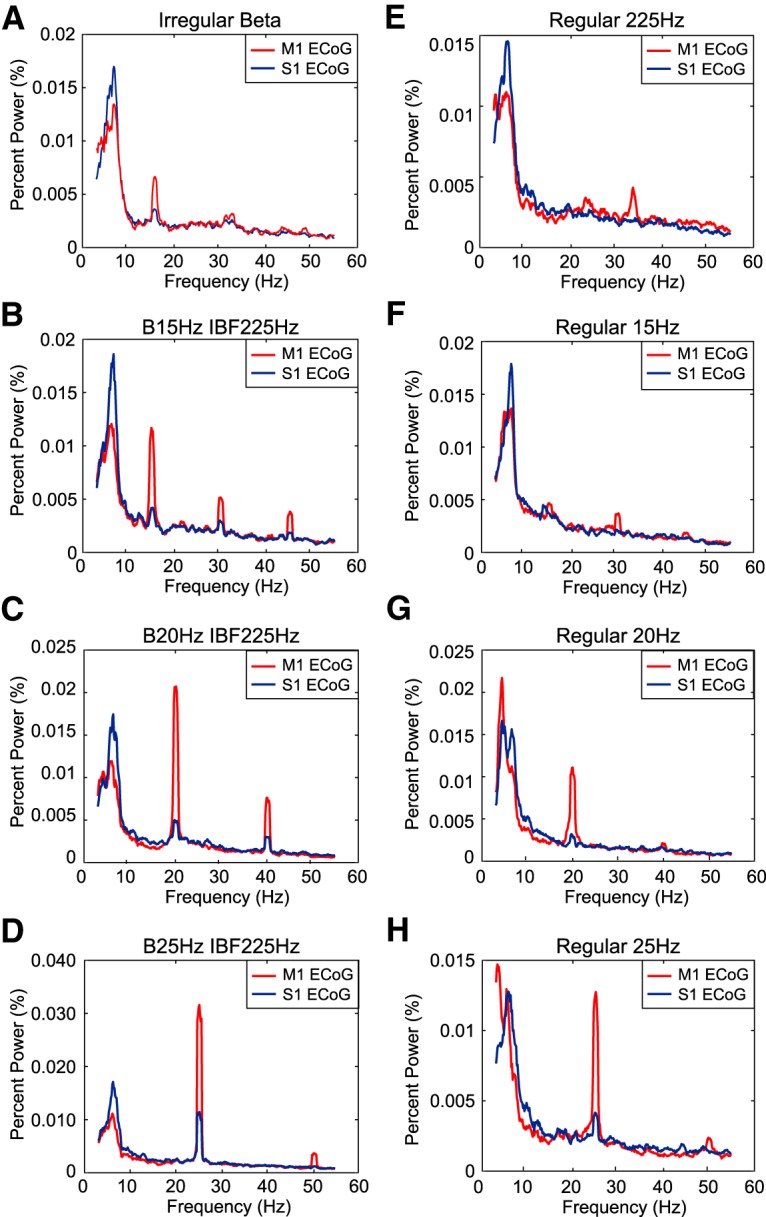
Effect of STN stimulation patterns on ipsilateral M1 and contralateral S1 ECoG. ***A–H***, Multi-taper spectra normalized to percentage of total power for a representative animal across stimulation patterns at the maximum stimulation amplitude for that animal.

**Figure 15.**
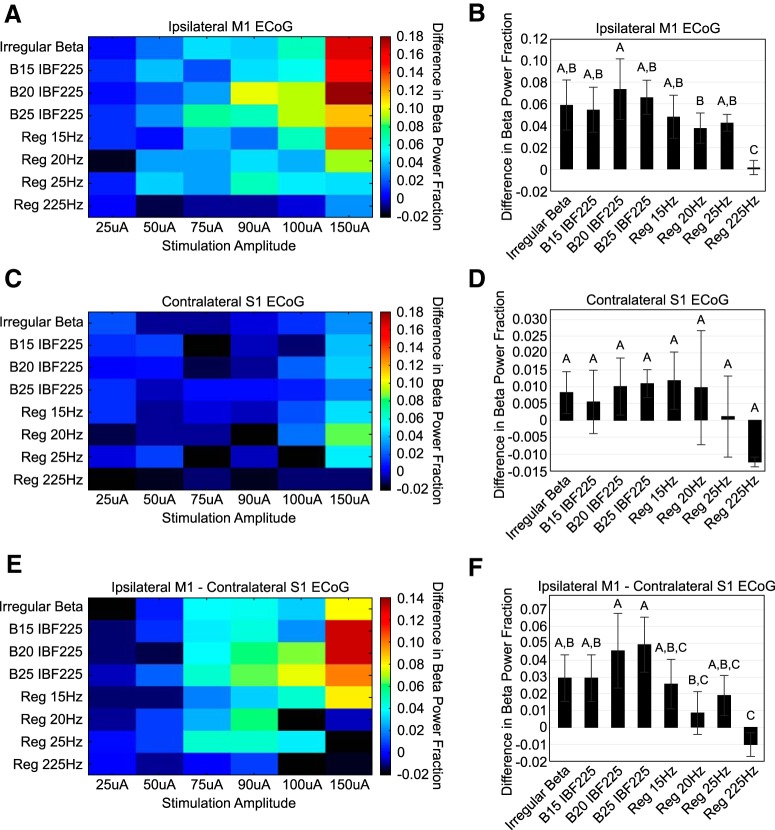
Effect of STN stimulation patterns on ipsilateral M1 and contralateral S1 ECoG. ECoG data from *n* = 10 rats were analyzed. Patterns not sharing the same letter are significantly different from each other (*p* < 0.05). Error bars indicate mean ± SE. ***A***, Difference in β power fraction between ipsilateral M1 ECoG during stimulation and ipsilateral M1 ECoG before stimulation. Two-way ANOVA performed on the difference in β power fraction found a significant effect of pattern (*p* < 0.0001), amplitude (*p* < 0.0001), and pattern × amplitude (*p* = 0.0004). ***B***, *Post hoc* Tukey’s test results for factor pattern across amplitude for difference in β power fraction for ipsilateral M1 ECoG. A significant difference was found between B20 IBF225 Hz and regular 20-Hz stimulation (*p* = 0.04). All patterns were significantly different from regular 225-Hz stimulation. ***C***, Difference in β power fraction between contralateral S1 ECoG during stimulation and contralateral S1 ECoG before stimulation. Two-way ANOVA performed on the difference in β power fraction found a significant effect of amplitude (*p* = 0.0008) but no significant effects of pattern (*p* = 0.49) or pattern × amplitude (*p* = 0.46). ***D***, *Post hoc* Tukey’s test results for factor pattern across amplitude for difference in β power fraction for contralateral S1 ECoG. No statistically significant differences across pattern were found (*p* < 0.05). ***E***, Difference in β power fraction between ipsilateral M1 ECoG and contralateral S1 ECoG. Two-way ANOVA found a significant effect of pattern (*p* = 0.0002), amplitude (*p* < 0.0001), and pattern × amplitude (*p* < 0.0001). ***F***, *Post hoc* Tukey’s test for factor pattern across amplitude for difference in β power fraction between ipsilateral M1 ECoG and contralateral S1 ECoG. Multiple significant differences were found between burst patterns and some continuous frequency patterns (*p* < 0.05).

We quantified the effect of STN stimulation on ipsilateral M1 and contralateral S1 ECoG to determine whether stimulation was generating BFP at multiple points within the cortico-basal ganglia loop. The effect of STN stimulation on ECoG activity is displayed in Figure 14 for a representative animal and summarized in Figure 15. Figure 15*A* displays the difference in β power fraction between ipsilateral M1 ECoG during stimulation and ipsilateral M1 ECoG before stimulation. The difference in β power fraction increased with amplitude, and a significant effect of pattern and a significant interaction term were found (Table 9; Fig. 15*A*). Most patterns generated equivalent amounts of BFP in ipsilateral M1 ECoG as compared to the pre-stimulation baseline. However, the B20 IBF225 Hz pattern generated a significantly greater increase in BFP than regular 20-Hz continuous stimulation (*p* = 0.04, Tukey’s HSD), and all patterns generated a significantly greater increase in BFP than the continuous high-frequency pattern (*p* < 0.05), which did not generate an amount of BFP that was different from zero (Fig. 15*B*).

**Table 9.** Effect of STN stimulation patterns on ECoG activity: results of statistical analyses for effects on ipsilateral M1 and contralateral S1 ECoG

Metric	Parameter	*n*/group	Test statistic	df	*p* value
Ipsilateral M1 ECoG					
		Two-way ANOVA (*F*)			
Difference in BPF	Amplitude	*n* = 10	161.7	1,0.056	<0.0001
	Stim pattern		8.50	7,0.020	<0.0001
	Interaction		5.28	7,0.013	0.0004
Contralateral S1 ECoG					
		Two-way ANOVA (*F*)			
Difference in BPF	Amplitude	*n* = 10	13.7	1,0.006	0.0008
	Stim pattern		0.95	7,0.003	0.49
	Interaction		0.99	7,0.003	0.46
Ipsilateral M1 ECoG - contralateral S1 ECoG					
		Two-way ANOVA (*F*)			
Difference in BPF	Amplitude	*n* = 10	52.0	1,0.019	<0.0001
	Stim pattern		5.84	7,0.015	0.0002
	Interaction		7.25	7,0.019	<0.0001

BPF = β power fraction.

Figure 15*C* displays the difference in β power fraction between contralateral S1 ECoG during stimulation and contralateral S1 ECoG before stimulation. While there was a significant effect of amplitude, the increase in β power fraction with stimulation was not significant (Table 9; Fig. 15*C*).

The difference in BFP between ipsilateral M1 ECoG during stimulation and contralateral S1 ECoG during stimulation as a function of stimulation pattern and amplitude is displayed in Figure 15*E*. The difference in β power fraction increased significantly with amplitude (Table 9; Fig. 15*E*). Also, as in SNr units, there was a significant effect of pattern with a significant interaction term (Table 9; Fig. 15*E*). *Post hoc* Tukey’s test showed a tendency toward burst patterns causing greater increases in BFP in ipsilateral M1 ECoG versus contralateral S1 ECoG during stimulation. In particular, the B20 IBF 225 Hz and the B25 IBF225 Hz patterns increased BFP in ipsilateral M1 ECoG relative to contralateral S1 ECoG to a greater degree than the regular 20 Hz (B20 IBF 225 Hz vs regular 20 Hz: *p* = 0.04; B25 IBF225 Hz vs regular 20 Hz: *p* = 0.02) and regular 225 Hz (B20 IBF 225 Hz vs regular 225 Hz: *p* = 0.0006; B25 IBF225 Hz vs regular 225 Hz: *p* = 0.0002) patterns (Fig. 15*F*). The B15 IBF225 Hz and Irregular β patterns also increased this difference in BFP to a greater degree than the regular 225 Hz (B15 IBF225 Hz vs regular 225 Hz: *p* = 0.03; irregular β vs regular 225 Hz: *p* = 0.03) pattern (Fig. 15*F*). These data are consistent with the results of the computational model and the results of the SNr unit analysis, demonstrating that β-patterned paradigms increase BFP at multiple sites in the cortico-basal ganglia circuit and do so more effectively than constant frequency stimulation.

### Post-6-OHDA lesion single unit recordings

**Figure 16.**
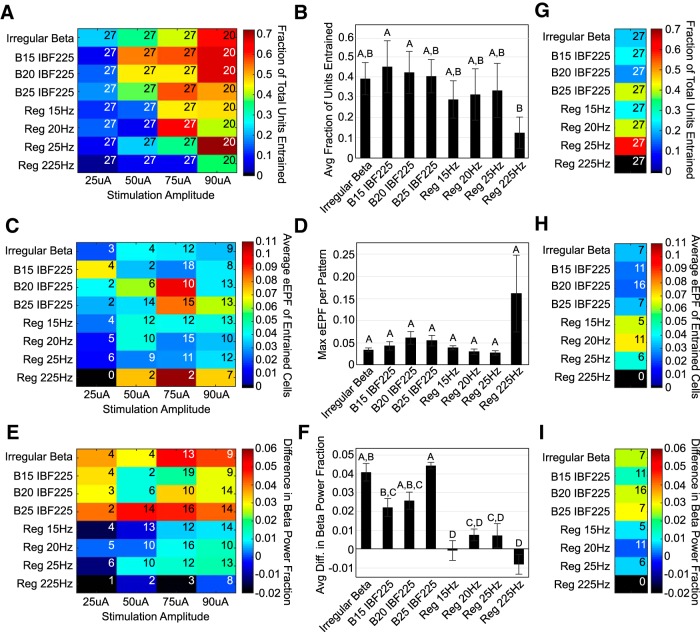
Effect of STN stimulation patterns on eEPF in 6-OHDA**-**treated animals. Patterns not sharing the same letter are significantly different from each other (*p* < 0.05). Error bars indicate mean ± SE. ***A***, Fraction of total SNr units entrained as a function of stimulation pattern and amplitude in 6-OHDA-lesioned animals not pre-treated with levodopa. Inset numbers refer to total number of units assessed (*n*) per stimulation pattern and amplitude setting. Two-way ANOVA performed on entrainment fractions found a significant effect of pattern (*p* = 0.032) and amplitude (*p* < 0.0001), but no significant pattern × amplitude interaction (*p* = 0.91). ***B***, *Post hoc* Tukey’s test results for factor pattern for entrainment fraction ANOVA in 6-OHDA-lesioned animals not pre-treated with levodopa. Statistically significant differences between regular 225 Hz and B15IBF225/B20IBF225 were found (*p* < 0.05). ***C***, Average eEPF of entrained SNr units as a function of stimulation pattern and amplitude in 6-OHDA-lesioned animals not pre-treated with levodopa. Inset numbers refer to total number of entrained units (*n*) included in the calculation. Two-way ANOVA found no significant effect of amplitude (*p* = 0.52), pattern (*p* = 0.35), or pattern × amplitude (*p* = 0.99). ***D***, *Post hoc* Tukey’s test results for factor pattern for average eEPF value in 6-OHDA-lesioned animals not pre-treated with levodopa. No significant differences were found among patterns (*p* < 0.05). ***E***, Difference in β power fraction between stimulation and pre-stimulation power spectra as a function of stimulation pattern and amplitude in 6-OHDA-lesioned animals not pre-treated with levodopa. Inset numbers refer to total number of entrained units (*n*) included in the calculation. Two-way ANOVA found a significant effect of pattern (*p* < 0.0001) and amplitude (*p* = 0.021) but no significant pattern × amplitude interaction (*p* = 0.53). ***F***, *Post hoc* Tukey’s test results for factor pattern for difference in β power ANOVA. Patterns not connected by the same letter are significantly different from each other (*p* < 0.05). ***G***, Fraction of total SNr units entrained as a function of stimulation pattern in 6-OHDA-lesioned animals pre-treated with levodopa. Inset numbers refer to total number of units assessed (*n*) per stimulation pattern and amplitude setting. Two-way ANOVA comparing entrainment fraction with levodopa pre-treatment to the average of the entrainment fractions without levodopa at 75 and 90 μA (***A***) found a significant effect of drug (*p* = 0.023) but no significant effect of pattern (*p* = 0.65) or drug × pattern (*p* = 0.60). *Post hoc* Student’s *t* test revealed a greater fraction of units entrained without levodopa than with the application of levodopa (*p* = 0.023). ***H***, Average eEPF of entrained SNr units as a function of stimulation pattern in 6-OHDA-lesioned animals pre-treated with levodopa. Inset numbers refer to total number of entrained units (*n*) included in the calculation. Two-way ANOVA comparing average eEPF with levodopa pre-treatment to the average of eEPF values without levodopa at 75 and 90 μA (***B***) found no effect of drug (*p* = 0.69), pattern (*p* = 0.64), or drug × pattern (*p* = 0.19). ***I***, Difference in β power fraction between stimulation and pre-stimulation power spectra as a function of stimulation pattern in 6-OHDA-lesioned animals pre-treated with levodopa. Inset numbers refer to total number of entrained units (*n*) included in the calculation. Two-way ANOVA comparing average difference in β power fraction with levodopa to the average difference in β power fraction without levodopa at 75 and 90 μA (***C***) found a significant effect of drug (*p* = 0.0021), and pattern (*p* = 0.0002), but no significant pattern × drug interaction (*p* = 0.18). *Post hoc* Student’s *t* test for factor drug found a smaller increase in β power fraction with levodopa than without levodopa (*p* = 0.0021). *Post hoc* Tukey’s test for factor pattern found that β-patterned paradigms, particularly B25 IBF225, B20 IBF225, and irregular β patterns, were superior to increasing β power fraction than low**-**frequency control patterns.

In 6-OHDA-lesioned rats not treated with levodopa, the effect of stimulation pattern and amplitude on fraction of units entrained, average eEPF, and change in β power fraction was largely similar to that seen in intact animals (Fig. 16). In 6-OHDA-lesioned animals not pre-treated with levodopa, significant effects of amplitude and pattern on entrainment fraction were found with no significant interaction term (Table 8; Fig. 16*A*). *Post hoc* Tukey’s test found significant differences between B15 IBF225 Hz and regular 225 Hz (*p* = 0.02) and B20 IBF225 Hz and regular 225 Hz (*p* = 0.04; Fig. 16*B*). Average eEPF was not affected by amplitude or pattern (Table 8; Fig. 16*C*). Significant effects of amplitude and pattern were observed on the difference in β power fraction in SNr units without a significant interaction term (Table 8; Fig. 16*E*). *Post hoc* Tukey’s testing showed statistically significant differences between B25 IBF225 Hz and patterns regular 225 Hz (*p* < 0.0001), regular 15 Hz (*p* < 0.0001), regular 25 Hz (*p* = 0.0002), regular 20 Hz (*p* = 0.0002), and B15 IBF 225 Hz (*p* = 0.02; Fig. 16*F*). Additional significant differences were found between irregular β and patterns regular 225 Hz (*p* < 0.0001), regular 15 Hz (*p* < 0.0001), regular 25 Hz (*p* = 0.0002), and regular 20 Hz (*p* = 0.0002; Fig. 16*F*). Finally, pattern B20 IBF225 Hz was significantly different from regular 225 Hz (*p* = 0.0023) and regular 15 Hz (*p* = 0.0062), and pattern B15 IBF225 Hz was significantly different from regular 225 Hz (*p* = 0.0065) and regular 15 Hz (*p* = 0.02; Fig. 16*F*). Thus, overall, in 6-OHDA-lesioned animals not pre-treated with levodopa, entrainment fraction and average eEPF largely were equivocal across patterns (Fig. 16*B*,*D*), but β-patterned paradigms were superior to continuous low-frequency paradigms at increasing SNr unit BFP, and continuous high-frequency stimulation suppressed β power (Fig. 16*F*). Comparing across Figures 13, 16 using a multi-way ANOVA on factors amplitude, pattern, and lesion state, in addition to significant effects of amplitude and pattern, significant effects of lesion state on entrainment fraction (*p* < 0.0001, multi-way ANOVA, $F_{\left(1,0.40\right)}$ = 52.2) and change in β power fraction (*p* < 0.0001, multi-way ANOVA, $F_{\left(1,0.005\right)}$ = 21.6) were found. No effect of lesion state on average eEPF was found (*p* = 0.41). *Post hoc* Student’s *t* test revealed that SNr units were more readily entrained in 6-OHDA-lesioned animals (*p* < 0.0001; Figs. 13*A*,*B* vs 16*A*,*B*) but that more β power was induced in intact animals as compared to 6-OHDA-lesioned animals (*p* < 0.0001; Figs. 13*E*,*F* vs 16*E*,*F*). The interpretation of these statistical differences between intact and 6-OHDA-lesioned non-levodopa-treated rats should be viewed with the caveat of a smaller sample of units analyzed in 6-OHDA-lesioned animals than in intact animals.

Similar but weaker trends in SNr unit response to STN stimulation were seen in 6-OHDA-lesioned animals treated with levodopa (Fig. 16*G-I*). Entrainment fraction, average eEPF, and change in β power fraction were compared across pattern and drug state in 6-OHDA-lesioned rats. Statistically significant effects of drug state were found on entrainment fraction and β power fraction, which both decreased in 6-OHDA-lesioned rats in response to levodopa (Table 8; Fig. 16*G-I*). A statistically significant effect of pattern was not found for entrainment fraction but was found for β power fraction (Table 8; Fig. 16*G-I*). However, *post hoc* Tukey’s test showed that β-patterned paradigms remained superior to low-frequency paradigms in amplifying β power fraction in SNr units of 6-OHDA-lesioned animals treated with levodopa. In particular, B25 IBF225 Hz was significantly different from patterns regular 20 Hz (*p* = 0.001), regular 15 Hz (*p* = 0.0014), regular 25 Hz (0.0027), and B15 IBF225 Hz (*p* = 0.02; Fig. 16*I*). Pattern irregular β was significantly different from patterns regular 20 Hz (*p* = 0.001), regular 15 Hz (*p* = 0.0014), regular 25 Hz (*p* = 0.0027), and B15 IBF225 Hz (*p* = 0.022; Fig. 16*I*). Finally, pattern B20 IBF225 Hz was significantly different from patterns regular 20 Hz (*p* = 0.0064), regular 15 Hz (*p* = 0.01), and regular 25 Hz (*p* = 0.022; Fig. 16*I*).

## Discussion

A spectrum of data supports a correlation between β frequency activity and bradykinesia and akinesia in PD, but evidence of causality is lacking. We investigated whether a causal link exists by applying novel patterns of stimulation that mimic the β frequency activity present in STN in PD to healthy animals to determine whether they consequently exhibited parkinsonian symptoms. Single unit and ECoG recordings demonstrated that the patterns of STN stimulation entrained SNr unit activity and increased SNr unit and ipsilateral M1 ECoG BFP; β-patterned stimulation paradigms were particularly effective. However, we did not detect changes in motor behavior with the applied stimulation across five validated measures of motor activity. The application of betaergic stimulation to 6-OHDA-lesioned animals treated with levodopa to disrupt endogenous β frequency activity similarly did not cause discernible changes in motor symptoms, suggesting that the parkinsonian neural substrate does not amplify the behavioral effects of induced β activity introduced via the STN. Our results suggest that STN β frequency oscillations (BFOs) are not sufficient for the generation of bradykinetic/akinetic symptoms in PD.

### Effect of β-patterned stimulation on motor and neural activity in intact animals

Betaergic STN stimulation in healthy rats did not result in detectable worsening of motor performance metrics in our behavioral tasks. The skilled forelimb lever press test detected significant decreases from baseline in total trials attempted with some β-patterned paradigms. However, the modest scale of these reductions and the lack of a corresponding decline of other performance metrics in this task suggests that this finding is not reflective of an induction of akinetic symptoms, particularly when compared to the consequential reductions in performance resulting from 6-OHDA lesion. We used a battery of validated behavioral tests to quantify different metrics of forelimb and body bradykinesia. Three of five tasks–the bar test, adjusting steps test, and skilled forelimb lever press test–were sensitive to changes in performance after 6-OHDA lesion. Further, a minimum of five rats were used in these experiments, and given the effect sizes seen with 6-OHDA lesioning in Figures 6, 8, 10, these behavioral tasks were powered to detect statistically significant differences between 6-OHDA and healthy rat performance with a power ≥0.80. The bar test and adjusting steps tests in particular were powered with *n* ≥ 5 rats (≥0.80) to detect effect sizes due to betaergic stimulation as small as 33% and 50% of the effect sizes caused by of 6-OHDA lesioning. Therefore, our study design was sufficiently powered to detected effect sizes that were meaningful and interesting for our hypothesis, and we acknowledge that more subtle deficits due to betaergic stimulation may have gone undetected. The open field test and forelimb use asymmetry tests did not detect changes in performance after 6-OHDA lesion. These tests are “passive” rather than “active” tasks and require that the animal is interested in exploring the test chamber, which is difficult to maintain over multiple trials. The 6-OHDA lesion state thus represented an important control in our behavioral studies, and these differences across tasks highlight the strength of our experimental design in not relying on a single behavioral metric to assess our hypothesis.

As in the computational model, β-patterned STN stimulation paradigms effectively entrained and increased β frequency activity in SNr units and ipsilateral M1 ECoG in intact animals in a manner that increased with stimulation amplitude. Regarding the effect on SNr unit activity, all β-patterned and low-frequency stimulation patterns equally entrained unit activity and did not differ significantly in average eEPF. These findings indicate that β-patterned and low-frequency stimulation captured and drove downstream neural activity equivalently. However, β-patterned stimulation paradigms were superior to low-frequency stimulation at inducing BFP. This finding supports our premise that bio-inspired stimulation patterns are better suited to driving β frequency activity than low-frequency stimulation patterns. Although eEPFs of 4.8–10.6% may seem small, these values align with those calculated by Agnesi et al. (2015; 8.7 ± 8.4%) for behaviorally effective high-frequency STN DBS in MPTP**-**treated rhesus monkeys, implying that the applied stimulation paradigms capture SNr unit activity in a manner similar to clinically effective DBS. Additionally, the observed latencies of peak entrainment for β-patterned and low-frequency stimulation paradigms were consistent with studies of EPSC latencies between STN and SNr in rat brain slices. STN stimulation evoked complex EPSCs in the SNr consisting of an early monosynaptic current occurring at 4.6 ± 0.3 ms and variable, likely polysynaptic, later currents that were postulated to have been generated by stimulation of STN recurrent axon collaterals (Shen and Johnson, 2006). The latency at which the first peak of entrainment occurred for all patterns was consistent with the latency of this early monosynaptic current. The consistency of our results with these other studies supports the assertion that our β-patterned STN stimulation paradigms were indeed generating β frequency neural activity. One limitation of the eEPF calculation in assessing SNr unit entrainment to stimulation is that shorter IPIs reduce the probability of detecting a spike within that window. The regular 225-Hz pattern performed worse at entraining SNr units according to this metric as the IPI comprised 4.4 ms. When this pattern did entrain units, it generated spikes in a manner comparable to that of the other patterns and effectively suppressed BFP as predicted by the computational model.

The betaergic patterns of STN stimulation used in the present study were delivered continuously and without reference to the phase of the endogenous β activity. Cagnan et al. (2017) demonstrated that DBS of the thalamus produced differential effects on tremor in patients with essential tremor, dependent on the phase of the tremor cycle when the (burst of) stimulation was delivered. However, the relationship between the stimulation bursts and the phase of tremor wandered during continuous stimulation, and the phase of tremor when any particular burst of stimulation was delivered was variable. Therefore, given the comparatively long epochs of stimulation that we employed, we expect that the bursts of betaergic stimulation wandered across the entire range of phase of any intrinsic β activity. As our *in vivo* recordings demonstrated induction of substantial β-bad oscillatory activity, we do not expect that phase-dependent stimulation would yield results different from those reported here.

Similar effects were seen on the ECoG activity. Stimulation patterns significantly increased β power fraction in ipsilateral M1 ECoG as compared to the pre-stimulation baseline. However, the B20 IBF225 Hz pattern was superior in generation of BFP than its corresponding low-frequency control pattern. Contralateral S1 ECoG served as a control. While ipsilateral projections between S1 and STN have been seen in a rat histologic study, no evidence of a connection between contralateral S1 and STN was found (Canteras et al., 1988). Comparing the results in ipsilateral M1 and contralateral S1 provides a sense of how much BFP in the calculated spectra is due to the lingering effects of stimulation artifact after data processing (contralateral S1) versus a true physiologic effect of stimulation (ipsilateral M1). The effects of stimulation on BFP in contralateral S1 ECoG were not significantly different across pattern and not much different from zero. Relating ipsilateral M1 ECoG spectral power results to contralateral S1 spectral power results during stimulation further enhanced the difference seen across patterns with B20 IBF225 Hz and B25 IBF225 Hz patterns demonstrating a significantly greater increase in BFP than the low-frequency regular 20-Hz pattern. These results, in combination with the lack of bradykinetic/akinetic symptom generation in any motor task, further indicate a lack of a causal relationship between STN BFO and bradykinesia/akinesia in PD.

### Effect of β-patterned stimulation on motor and neural activity in 6-OHDA-lesioned animals

As in intact animals, performance in the adjusting steps and skilled forelimb reaching tests were unaffected in a discernable manner by β-patterned STN stimulation in levodopa-treated 6-OHDA-lesioned rats. In both tasks, 130-Hz stimulation and injection of levodopa significantly improved motor performance highlighting the sensitivity of the task to changes in bradykinetic/akinetic symptoms. The animals, however, continued to perform at a high level with β-patterned STN stimulation.

In 6-OHDA-lesioned rats not treated with levodopa, β-patterned STN stimulation paradigms were largely as effective at entraining and increasing β frequency activity in SNr units as in intact rats. Similarly, while all β-patterned and low-frequency stimulation patterns equally entrained unit activity and did not differ in average eEPF, β-patterned stimulation was superior to low-frequency controls in increasing β frequency activity in SNr units in untreated 6-OHDA-lesioned animals. Injection of levodopa weakened stimulation-induced unit entrainment and induction of BFP. Levodopa reduces β band synchrony in PD patients and 6-OHDA-lesioned rats (Levy et al., 2002). A potential limitation of our study design is that rather than simply disrupting endogenous β oscillatory activity, the effects of levodopa may have counteracted the ability to induce artificial synchrony as strongly as in intact and non-levodopa-treated 6-OHDA-lesioned animals. Furthermore, the same lesioned animal cohort participated in SNr unit recordings with and without levodopa treatment; channel wave form comparisons confirmed that the units analyzed in both lesion studies were largely identical (data not shown). These observations suggest that a true biological effect lay behind the effect of drug state in the comparative analysis of entrainment of SNr unit activity in 6-OHDA-lesioned animals with and without levodopa treatment. Our goal in applying the same methodology to intact animals and to levodopa-treated 6-OHDA-lesioned animals was to see if the inherent changes in neural circuitry in PD would amplify or unmask the effect of our betaergic patterns. However, induction of BFP in SNr unit activity in 6-OHDA-lesioned animals not treated with levodopa did not exceed, and was slightly less than, that seen in intact animals.

In summary, we did not detect a causal link between STN β frequency activity and bradykinesia/akinesia in PD within the limits of our experimental design. Other studies have pursued a similar objective but focused on introducing continuous low-frequency STN stimulation at β band frequencies in intact or PD subjects and assessing potential deleterious effects on motor performance (Chen et al., 2007; Pogosyan et al., 2009, Syed et al., 2012). We designed unique bursting patterns of stimulation intended to mimic the activity seen in STN cells in PD to amplify β frequency activity, and applied them, along with continuous low-frequency stimulation, to the STN of intact and 6-OHDA-lesioned rats. We confirmed that our patterns entrained neural activity in downstream nuclei *in vivo* as compared to other studies that demonstrate this phenomenon *in vitro* (Syed et al., 2012) and confirmed entrainment of neural activity in the same animals that performed the motor tasks. While low-frequency stimulation and the novel bursting patterns equally entrained SNr units, the novel bursting patterns were superior at amplifying β frequency activity as compared to the low-frequency stimulation used in prior studies. Yet, we detected no impact on motor performance despite use of an extensive battery of behavioral tasks to assess forelimb bradykinesia/akinesia that were sensitive enough to detect deterioration in motor performance following 6-OHDA lesioning with a minimum of three rats. We do not claim to have proven that a link between STN β frequency activity and bradykinesia/akinesia in PD does not exist. Indeed, such a claim cannot be proved, and we acknowledge several limitations of our study. The artificial β band power we generated may be fundamentally different from the broad band endogenous β power that emerges across neural circuits in 6-OHDA rats and patients with PD. Additionally, it is unclear from studies of parkinsonian models and PD patients what magnitude of BFP would be expected to correlate with a debilitating degree of bradykinesia for which the subject could not compensate. Finally, while we assessed motor performance in a variety of tasks, the majority of these tasks measure gross body or limb bradykinesia/akinesia, and different behavioral tasks aimed at quantifying more subtle metrics of limb bradykinesia may have yielded evidence of induced deficits.

Given these limitations, while our approach was novel, our results do concur with those of other groups that found no (Syed et al., 2012) or slight (Chen et al., 2007; Pogosyan et al., 2009) effects on motor performance of imposed STN β frequency activity in pre-clinical and clinical studies. Our results complement the existing body of work exploring the relationship of STN β frequency activity and bradykinesia/akinesia in PD, which call into question this assumed correlative link between BFO and bradykinesia in PD. Elucidating the pathophysiology of PD motor symptoms and the origin of BFO will impact the understanding of the field and the effectiveness of treatment options.

## Synthesis

Reviewing Editor: Alfonso Araque, University of Minnesota Decisions are customarily a result of the Reviewing Editor and the peer reviewers coming together and discussing their recommendations until a consensus is reached. When revisions are invited, a fact-based synthesis statement explaining their decision and outlining what is needed to prepare a revision will be listed below. The following reviewer(s) agreed to reveal their identity: Nicole Swann. Note: If this manuscript was transferred from JNeurosci and a decision was made to accept the manuscript without peer review, a brief statement to this effect will instead be what is listed below.

The reviewers have found merit and interest on the findings reported. I concur with the reviewers' comments expressing the interest of the study. However, they also expressed some concerns about several issues that need to be addressed and clarified. I also consider pertinent the concerns expressed. I believe that addressing those concerns, which will definitely improve the manuscript, is necessarily required to firmly validate the results and support the conclusions.

Specific comments of the reviewers:

Reviewer 1.

Summary:

The authors investigated whether the canonical beta oscillations that occur in PD were necessary and sufficient for bradykinesia in rats. This was done in a very ingenious way by constructing beta-patterned signals from a biophysical model. These signals as well as several other variants were applied to a multitude of behavioral experiments, where most were shown to cause nonsignificant changes to behavior.

I understand the authors had a different hypothesis going in, (ie looking for significant results but found none). However, this does not prove the converse is true. I applaud the authors for taking the right approach by conducting more experiments to satisfy the concerns of all readers but with the lack of statistical significance with the low sample sizes, it still does not inspire confidence in their conclusions.

There are low sample sizes on all the experiments (Table 1). And when the p-values are not significant, the authors claim there is no effect. With the low sample size and the possible nonnormality of the data, it may be difficult to prove (or disprove significance) without further justification or more statistical tools (confidence intervals).

Pros

* Very careful study, and very well done from an experimental standpoint.

* Interesting results, that if true, could be very important: high significance.

* Use of a biophysical model to promote experiments, is always a satisfying approach.

* In vivo results expand the literature from classic in vitro experiments.

Cons

* Too long. This paper is huge and unfortunately, that is not a good thing. It make sit very hard to read, let along interpret the nonsignificant results.

* The experiments were conducted with very low sample sizes. Table 1 sums up just low small some of the groups actually are. I understand it is hard to get a large n in animal models but if the reader is to believe nonsignificant results to mean no effect, larger sample sizes are needed.

* The type of normality test is not stated. With the low n value, I find it difficult to believe that any of the data follows the z/t distribution. And when a nonparametric test is used, power is being sacrificed, making the already shaky statistical argument used here even worse off.

* Although the p value was not below significance, it does not prove that there is no effect, especially with such a low sample size. Confidence intervals would be helpful to see here, to make their argument more believable.

* Very little changes from last review (text in red):

o Agree with reviewer #1:

* Low n is not justified.

o Agree with reviewer #2:

* Limitations not clear enough in discussion and the new additions to the manuscript seem to not address this. This seems to either be an oversight I the revision or a purposeful attempt to avoid fixing a large problem with their study.

* Reviewer 2's argument that it is “hard to prove a negative” is correct. The authors took the right steps to show “negative” results, but the low n is hurtful to the arguments presented here, especially with some of the low p-val

Reviewer 2.

The authors have conducted a very careful and through study. In some ways, it could even be two studies: first they tested different stimulation patterns meant to induced beta oscillatory activity using a biophysically-based computational model of the thalamo-cortical basal ganglia loop and examined impact of the stimulation on the neural firing in the model. Next, they applied these stimulation patterns to both intact and parkinsonsonian rats and accessed the impact of downstream beta. Finally, they tested the rats with an exhaustive number of motor tasks. They showed that, despite standard PD therapies (L-dopa and DBS) improving motor function, induction of beta power did not cause motor impairment for any test.

It is a bit challenging for me to review this manuscript at this stage, given that I was not an original reviewer. The original reviewer's brought up some important points and, in some cases, the authors didn't really address them (they just disagreed). I hope the original reviewers will also have an opportunity to respond.

From my read, I do think this manuscript is important and should be published, but could benefit from some changes, which I have outlined below. Given that this is a re-review, I do not think these changes are necessarily mandatory, but I do think they would improve the manuscript.

1) It was unclear to me why the authors concluded that beta oscillations were not necessary or sufficient to induce bradykinesia in rats. I agree that they have done an excellent job showing that beta is not sufficient. However, it is not clear to me that they showed that it is not necessary. I believe this would require showing that beta was not increased in the context of increased bradykinesia, or something that that effect. Without some kind of demonstration that an animal can have bradykinesia/akinesia w/o beta, it is not clear to me that they can say that beta isn't necessary. Clarification would help.

2) I see that the author's added a schematic of the experimental protocol to Figure 1 (in response to a reviewer's comments). However, I still found it hard to wrap my head around exactly how everything was done (order of stimulation, order of behavioral testing, number of rats for each test, etc.). As a reader, I would have a much easier time with this paper if this information were depicted more clearly in Fig 1 as a more detailed schematic. This is especially important because the manuscript is so long. If the reader could get a sense of what they did in a figure it could help guide the reader through the paper.

3) Beta changes in Figure 2 are very hard to see. An inset would help.

4) Reviewer #1's, point 1, brought up an important point about sample size which the authors didn't really address (at least not that portion of it). It is hard to prove that an effect doesn't exist, and the authors did an impressive job thoroughly probing the motor behavior. But the results seem very variable in their plots and sometimes there is really minimal data for each test (n=3 animals). They have already done a lot of work on this question and their findings are pretty convincing, so I wouldn't suggest collecting more data, but I do think they should mention the possibility that some comparisons may have been underpowered as a limitation in the manuscript. Also, to be a bit more explicit about the n for each comparison, the authors could consider showing individual data points on their bar graphs or use a swarm plot or depict individual observations in some other way.

5) Another limitation that I think the authors should address is that assuming that artificially applied beta is the same as endogenous beta may not be a fair assumption. For instance, they do show impressive downstream beta increases with their manipulation, but it is a much more narrowband than (patho)physiological beta that has been observed in humans and animals. This is just one example, but there are of course other ways that an external stimulation may differ from one that arises biologically. I think it would be appropriate to mention this.

6) The photos of rats were not very helpful for actually understanding the task in most cases. Some kind of schematic/drawing of what the rats actually did would be more informative for the reader.
